# Impact of later trading hours for bars and clubs on alcohol-related ambulance call-outs and crimes in Scotland: a controlled interrupted time series study

**DOI:** 10.1136/bmjph-2025-003722

**Published:** 2026-04-27

**Authors:** Nurnabi Sheikh, Jim Lewsey, Ines Henriques-Cadby, Colin Angus, Francesco Manca, Houra Haghpanahan, Emma McIntosh, Gemma Mitchell, Megan Cook, Karen J Maxwell, Andrea Mohan, Isabelle Uny, Elaina Smith, James Nicholls, Carol Emslie, Rachel O’Donnell, Niamh Fitzgerald

**Affiliations:** 1 Health Economics and Health Technology Assessment (HEHTA), School of Health and Wellbeing, University of Glasgow, Glasgow, UK; 2 Department of Mathematics, The University of Manchester, Manchester, UK; 3 School of Medicine and Population Health, The University of Sheffield, Sheffield, UK; 4 Institute for Social Marketing & Health, University of Stirling, Stirling, UK; 5 Centre for Alcohol Policy Research, La Trobe University, Melbourne, Victoria, Australia; 6 Research Centre for Health, School of Health and Life Sciences, Glasgow Caledonian University, Glasgow, UK; 7 Faculty of Health, University of Dundee, Dundee, UK; 8 Health Improvement, NHS Greater Glasgow and Clyde, Glasgow, UK

**Keywords:** Public Health, Causality, Epidemiology

## Abstract

**Introduction:**

Alcohol-related harms are prevalent late at night, especially on weekends, when high levels of intoxication contribute to increased rates of injury and violence. Reducing or increasing alcohol trading hours late at night in bars and clubs is generally associated with reduced and increased harms, respectively. This study evaluates the impact of later alcohol trading hours in the Scottish cities of Aberdeen and Glasgow on alcohol-related ambulance call-outs and crimes. Under local policy changes, 38 bars in Aberdeen had trading hours extended between 1 and 3 h up to 3am, and 10 nightclubs in Glasgow had a 1-h extension to 4am.

**Methods:**

Following a natural experiment evaluation framework, we used a controlled interrupted time series design to compare outcomes before and after policy changes, from May 2015 to March 2020. The primary outcome was a count of total weekend night-time alcohol-related ambulance call-outs. Secondary outcomes included weekend night-time crimes.

**Results:**

In Aberdeen, the policy led to a significant relative increase of 11.4% (effect size=4.643; 95% CI 0.292 to 8.994; p=0.036) in alcohol-related ambulance call-outs, and 8.5% (effect size=3.442; 95% CI 0.239 to 6.645; p=0.035) in reported crimes, at weekend night-times compared with Edinburgh (control). Findings were not significant and robust across analyses for Glasgow.

**Conclusion:**

Later alcohol trading hours had a significant negative impact on alcohol-related ambulance call-outs and reported crimes in Aberdeen (where more premises had longer extensions) but not in Glasgow, suggesting the number, capacity and type of premises moderated outcomes. This is important for the design of future national and local licensing policies and regulations.

**WHAT IS ALREADY KNOWN ON THIS TOPIC**

- Prior research has shown a strong association between alcohol availability, including late-night trading hours, and alcohol-related harms. Systematic reviews report that reducing hours of alcohol sales is linked to decreased alcohol-related ambulance call-outs, assaults and road accidents, whereas later trading hours tend to intensify these alcohol-related harms. International studies, such as those conducted in the Netherlands, Norway and Australia, have provided empirical evidence that even modest reductions or extensions in trading hours can lead to significant decreases or increases in alcohol-related ambulance call-outs, crimes and emergency department visits.

**WHAT THIS STUDY ADDS**

- This study provides novel and robust evidence on the impact of later trading hours for bars and clubs on alcohol-related ambulance call-outs and reported crimes in Scotland, using a controlled interrupted time series (CITS) design. By focusing on local policy changes in both Aberdeen and Glasgow, where certain venues of different types were permitted to extend their alcohol trading hours, this research leverages a natural experiment to provide novel insights. Our evaluation provides new evidence on the importance of local contexts on the magnitude and timing of change in trading hours for informing future policymaking decisions and research.

**HOW THIS STUDY MIGHT AFFECT RESEARCH, PRACTICE OR POLICY**

- This study provides novel and robust evidence on the impact of later trading hours for bars and clubs on alcohol-related ambulance call-outs and reported crimes in Scotland, using a controlled interrupted time series (CITS) design. By focusing on local policy changes in both Aberdeen and Glasgow, where certain venues of different types were permitted to extend their alcohol trading hours, this research leverages a natural experiment to provide novel insights. Our evaluation provides new evidence on the importance of local contexts on the magnitude and timing of change in trading hours for informing future policymaking decisions and research.

## Introduction

 Alcohol consumption led to approximately 1.8 million deaths globally in 2021 and is a risk factor for over 200 diseases.^1 2^ Alcohol’s harms extend beyond those consuming alcohol, including in contributing to increased crime, violence, road accidents and various forms of injury.^3 4^ In Scotland, 1277 deaths from causes wholly attributable to alcohol were recorded in 2023, averaging 24 deaths weekly.^5^ Alcohol also poses a significant burden for the healthcare system in Scotland: in 2022/2023, there were 31 206 alcohol-specific hospital admissions,^6^ and in 2019, it was estimated that 16% of ambulance call-outs were alcohol-related.^7^ Generally, alcohol harms are associated with greater density of premises licensed to sell alcohol, as well as increased days and hours during which alcohol can legally be sold.^8^ Acute alcohol harms are particularly prevalent late at night (ie, 12am to 5am), especially on weekends, when higher levels of intoxication can contribute to increased rates of injuries and violence.^9^ In 2019, the highest concentration of alcohol-related ambulance call-outs in Scotland occurred between 9pm and 1am on weekends, whereas call-outs unrelated to alcohol were more frequent between 10am and 9pm.^7^

Reducing late-night trading hours for alcohol on-premises outlets is associated with reductions in alcohol harms.^10^ Considering extensions to permitted trading hours for alcohol in on-premises outlets, separate systematic reviews concluded that extensions generally increased the likelihood of assaults, injuries and drink-driving incidents.^11 12^ A 2009 review found evidence that extensions were associated with increased harm, while reductions were associated with a decrease.^13^ The review emphasised the need for well-controlled research to further verify these associations. Another review based on a small number of studies concluded that extensions of two or more hours per day significantly increased alcohol harms, although no strong association was found for shorter spanning extensions due to limited evidence.^14^

In the UK, three separate systems (one in England and Wales, one in Scotland and one in Northern Ireland) control the granting of licences required to legally sell alcohol. Both the systems in England/Wales and in Scotland were substantially reformed in the mid-2000s. The Licensing Act (Scotland) 2005 introduced a wholly reformed system empowering local government licensing boards to set local policy and make decisions on premises licensing, guided by five overarching objectives. These boards set local on-trade alcohol trading hours policy, but the legislation includes a presumption against 24-h licensing. Under this system, late night alcohol trading hours have expanded in recent years,^15^ but their impact has never been evaluated in Scotland. The Licensing Act (2003) also established a completely new local government-led system to manage licensing in England and Wales that created a general presumption in favour of granting premises licences, with no presumption against 24-h alcohol sales. The 2003 Act was underpinned by the view that the fixed closing times that predated the Act (most pubs closing at 11pm) contributed to overcrowding, violence and disorder and that staggered dispersal may reduce these issues.^16^ Studies on the impact of extended licensing in England and Wales have not found consistent evidence of their impact on violence;^15^ they used weaker before-and-after designs without considering a control series compared with those in other international studies.^13 16^ Using a more robust interrupted time series design, Humphreys and colleagues found no evidence of an overall increase in violent crime in the city of Manchester following the Act but a significant 36% increase between 3am and 6am.^17^ Although more robust than earlier studies, wider health outcomes were not considered, further evidencing the need for additional research.^17^ Another UK-based study reported small changes in the timing of self-reported drinking occasions due to the implementation of the Act but no evidence of changes in finish time variation, post-loading behaviour, late-night drinking or overall alcohol consumption.^18^ To our knowledge, no study in the UK has considered the impact of later trading hours on alcohol-related ambulance call-outs.

Under the system established by the 2005 Act, local Licensing Boards (made up of elected members of local government) in Aberdeen and Glasgow have in recent years changed their local policies to allow premises in certain licence categories to apply for, and be granted, permission to sell alcohol later at night. This study aims to evaluate the impact of these later trading hours for bars and clubs on alcohol-related ambulance call-outs and reported crimes in both cities.

## Methods

### The policy changes: later trading hours for bars and clubs in Glasgow and Aberdeen

In Glasgow, from 12 April 2019, 10 nightclubs, which met certain quality and operating standards, were granted permission by the Glasgow City Licensing Board to remain open and sell alcohol for an additional hour to 4am (instead of 3am). This initial 12-month pilot became standard policy in 2023. In Aberdeen, a policy was introduced that allowed a wider range of alcohol premises to apply for later trading hours, which had previously only been available to nightclubs. From March 2017 to October 2020, 38 bars/pubs in Aberdeen were granted permission to sell alcohol between 1 and 2 hours later at night, up to 3am. Nightclubs remained closed throughout the COVID-19 restrictions and were therefore unable to use the additional hour, while bars and pubs were permitted to reopen earlier and to use extended hours during some periods. These recent policy changes in Aberdeen and Glasgow serve as the basis for this natural experiment evaluation.

### Study design

We used a controlled interrupted time series (CITS) design to evaluate the later closing times, comparing outcomes before and after policy implementation (May 2015–March 2020) in Glasgow and Aberdeen with a control group. The policy change date was 12 April 2019 for Glasgow, and a gradual adoption of extended hours in Aberdeen was beginning in early 2017, being the ‘interruption’ in the time series.

### Data sources

We obtained all ambulance call-outs in Scotland from the Scottish Ambulance Service (SAS) and crime data from Police Scotland. Ambulance data included records such as call-out time, coordinates of location, patient demographics and a flag indicating whether a call-out was alcohol-related (algorithmically generated from free-text fields in call-out records), as previously developed with SAS.^7^ Crime data were gathered from Police Scotland using Scottish Government Justice Directorate crime codes. Two authors (NF and JL) scrutinised and selected crime codes they judged to be more likely to be associated with alcohol, in consultation with Police Scotland. These included assaults, drunkenness, driving under the influence of alcohol and antisocial behaviour offences (see online supplemental table A4). Variables included time of recording, crime type, local council name, incident date and coordinates of crime location.

### Outcomes

Primary outcome: total weekend night-time alcohol-related ambulance call-outs (time between Fridays 20:00 to 23:59, Saturdays 00:00 to 05:59 and 20:00 to 23:59, and Sundays 00:00 to 05:59). Secondary outcomes: total weekend night-time ambulance call-outs and reported crimes.

The outcomes were analysed as weekly counts. We also modelled population size-adjusted outcomes to check robustness of our analysis.

### Control selection

We developed a principled and structured framework for selecting the study control, shown in online supplemental figure A1 (with steps in online supplemental appendix 1.1 for alcohol-related ambulance call-outs and in online supplemental appendix 1.2 for reported crimes) and details in the published statistical analysis plan (SAP).^19^ Following this framework, among the potential 30 control candidates (Scottish council areas, apart from Glasgow City and Aberdeen City), the City of Edinburgh council area (Edinburgh) was identified as the most suitable control for Aberdeen and Glasgow.

### Measurement of policy exposure variable

Separate exposure variables were generated for Glasgow and Aberdeen. The dates on which premises were permitted to trade later were staggered over time in Aberdeen; in contrast, in Glasgow, the later trading hours came into effect from the same date for all premises. In Glasgow, a binary exposure variable taking the value ‘0’, for time periods before the implementation of the new trading hours (ie, 12 April 2019), and ‘1’ for time periods thereafter, was used. In Aberdeen, a numerical measure of exposure was developed based on the additional person-hours available weekly across all premises. This measure reflects the staggered granting of later licensed hours, taking values between 0 (no extended hours) and 1 (all additional person-hours had been adopted across participating premises). The potential additional person-hours for a given venue in a week was calculated by using the formula [(additional hours) × (the number of nights additional hours are permitted to be used in a week) × (capacity of the venue)]. Full details can be found in the SAP.^19^

### Confounding variables

We included data on per capita gross disposable household income, weather conditions (mean temperature, rainfall) and total number of on-premises alcohol outlets to account for potential time-varying confounding effects. Additional adjusting for COVID-19 lockdown for extended time series as a part of sensitivity analysis (imposing closure or restricted hours for alcohol premises) was done through dummy variables. We included dummy variables to account for public holidays, outliers and COVID-19 restrictions which meant that the premises with later hours were not open at certain times (see online supplemental appendix 2.2 for details). As noted earlier, the value of the COVID-19 dummy variable over time reflected that nightclubs were fully closed during COVID-19 restrictions, while bars and pubs remained open for some periods. Data sources for the confounding variables are provided in online supplemental appendix 2.2.1.

### Statistical analysis

Data were analysed using autoregressive integrated moving average (ARIMA) models for both primary and secondary outcomes following the CITS design. We modelled the outcome differences between the interventions and control over time, for example, differences between alcohol-related ambulance call-outs in Aberdeen and Edinburgh. We followed Box-Jenkins model selection steps (identification, estimation and diagnostic checking) to obtain the best ARIMA model that adequately captures the underlying patterns in the data and provides accurate estimation. First, we used an Augmented Dickey-Fuller test to establish that the stationarity (ie, constant mean and variance over time) assumption was met. Then, we used autocorrelation and partial autocorrelation functions to identify the terms of the autoregressive and moving average models. After that, from the candidate models, the best fit was chosen according to Bayesian and Akaike Information Criterions. We next tested the suitability of the fitted model by performing diagnostic checks to ensure the residuals presented white noise characteristics. Policy exposure variables were added to the model as covariates to estimate intervention effect sizes. To check the robustness of our findings, we examined the effect size across ARIMA models adjusting for all covariates, and for statistically significant covariates only.

Subgroup analyses to examine differential impacts by age, and sex, were conducted for the primary outcome only. Analyses were performed in Stata/MP V.17.0 and RStudio (V.4.2.0) using the *Synth* package.^20^

### Sensitivity analysis

We conducted sensitivity analyses by extending the time series to post-COVID-19 restriction periods until July 2022 in Scotland to assess the robustness of our estimates. Additionally, we modelled population size-adjusted outcome rates. To check the robustness of our estimation, we additionally created two binary exposure variables specific to Aberdeen: one similar to Glasgow on the introduction of the new policy, and a second to indicate when the policy reached at least half its ‘strength’, taking a value of ‘0’ when Aberdeen’s policy exposure variable is less than 0.5, and ‘1’ otherwise.

We also conducted Synthetic Control (SC) modelling (see details in online supplemental appendix 3). A separate SC for Aberdeen and Glasgow was created for the population-adjusted rates of alcohol-related ambulance call-outs (see online supplemental appendix 3.1 for Aberdeen and online supplemental appendix 3.2 for Glasgow) and reported crimes (see online supplemental appendix 3.3 for Aberdeen and online supplemental appendix 3.4 for Glasgow), applying various specifications of pre-intervention outcomes.^21^ Covariates such as per capita gross disposable household income and population-adjusted number of on-premises alcohol outlets were incorporated in the construction of the SC. To assess the validity of our SC models, we split the pre-intervention period into training and validation phases and compared MSPEs (across validation periods) for the different model specifications. Online supplemental appendices 3.1.1 and 3.2.1 present the model specifications and validity tests for alcohol-related ambulance call-outs in Aberdeen and Glasgow, respectively. Online supplemental appendixces 3.3.1 and 3.4.1 contain the model specifications and validity tests for reported crimes in Aberdeen and Glasgow, respectively. We performed in-space placebo tests and calculated frequentist (frequency-based) p values for the effect sizes generated through SC. Details of synthetic control analyses and sensitivity tests are provided in online supplemental appendix 3.1.2 (Aberdeen) and online supplemental appendix 3.2.2 (Glasgow) for alcohol-related ambulance call-outs, and in online supplemental appendix 3.3.2 (Aberdeen) and online supplemental appendix 3.4.2 (Glasgow) for reported crimes. Additionally, we performed time-series models (ARIMA) to the synthetic control series to check the robustness of the estimated effect size (see online supplemental tables A17 and A18). Finally, a falsification test was conducted, by generating a false policy exposure variable signalling exposure 1 year before the true intervention date.

## Results

Between 1 May 2015 and 20 March 2020, a total of 4765 alcohol-related ambulance call-outs occurred in Aberdeen, 15 024 in Glasgow and 10 407 in Edinburgh during weekend night-times (online supplemental table A1). A total of 4425 crimes were reported in Aberdeen, 14 734 in Glasgow and 9354 in Edinburgh during weekend night-times, more than 65% of which in each city were ‘common assaults’ (online supplemental tables A1 and A4). Detailed descriptive analyses are given in online supplemental tables A2, A3 and A5.

**Figure 1:**
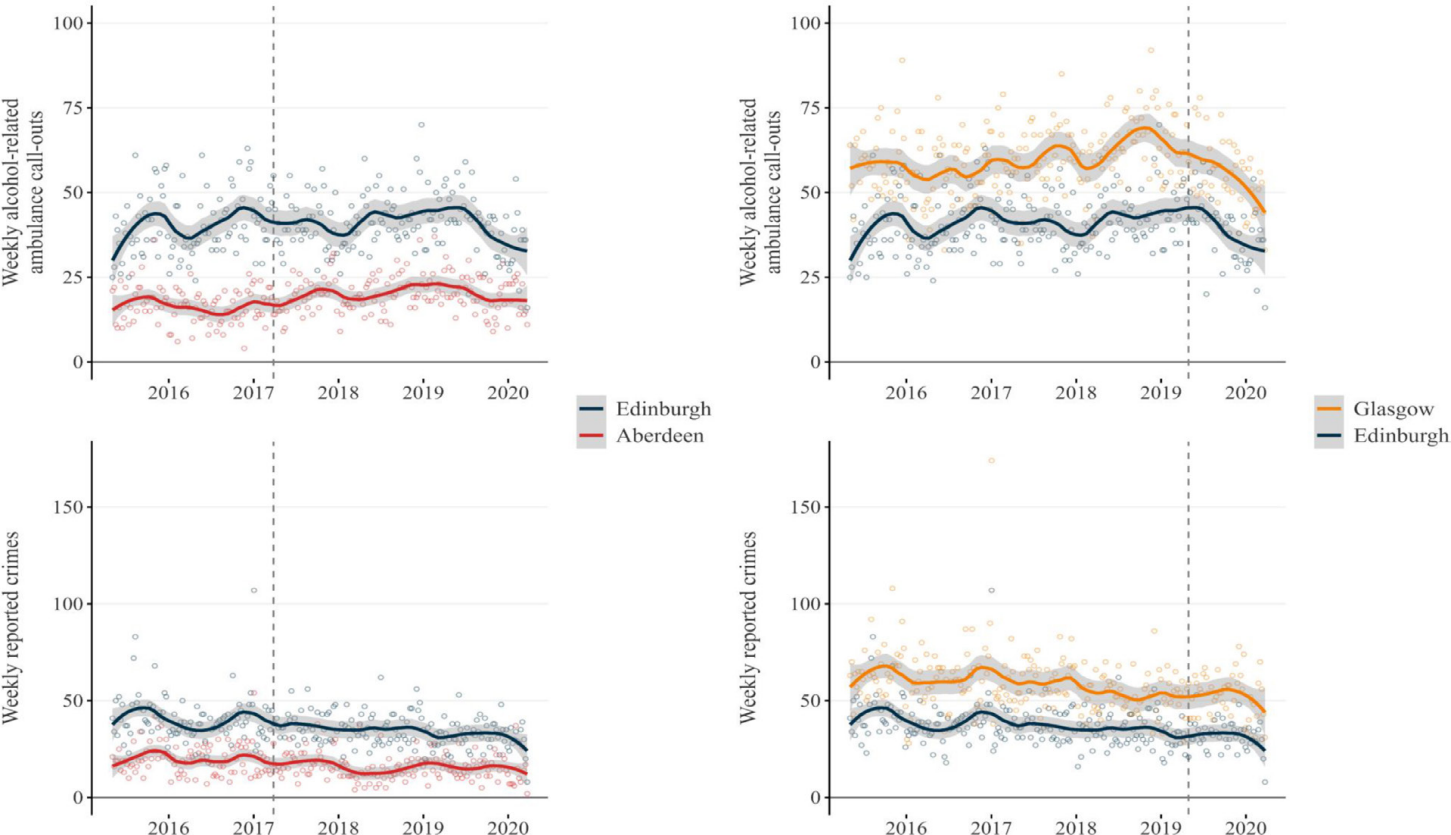
Number of weekend night-time alcohol-related ambulance call-outs and reported crimes over time in the intervention and control cities. Dots represent weekly data; solid lines indicate Locally Estimated Scatterplot Smoothing (LOESS)-smoothed trends and shaded areas represent 95% CIs; vertical dashed line represents start of intervention.

Prior to the policy changes, trends in alcohol-related ambulance call-outs and reported crimes between Aberdeen/Glasgow and Edinburgh were broadly comparable, with seasonal variation (figure 1 and online supplemental figure A3).

**Figure 2:**
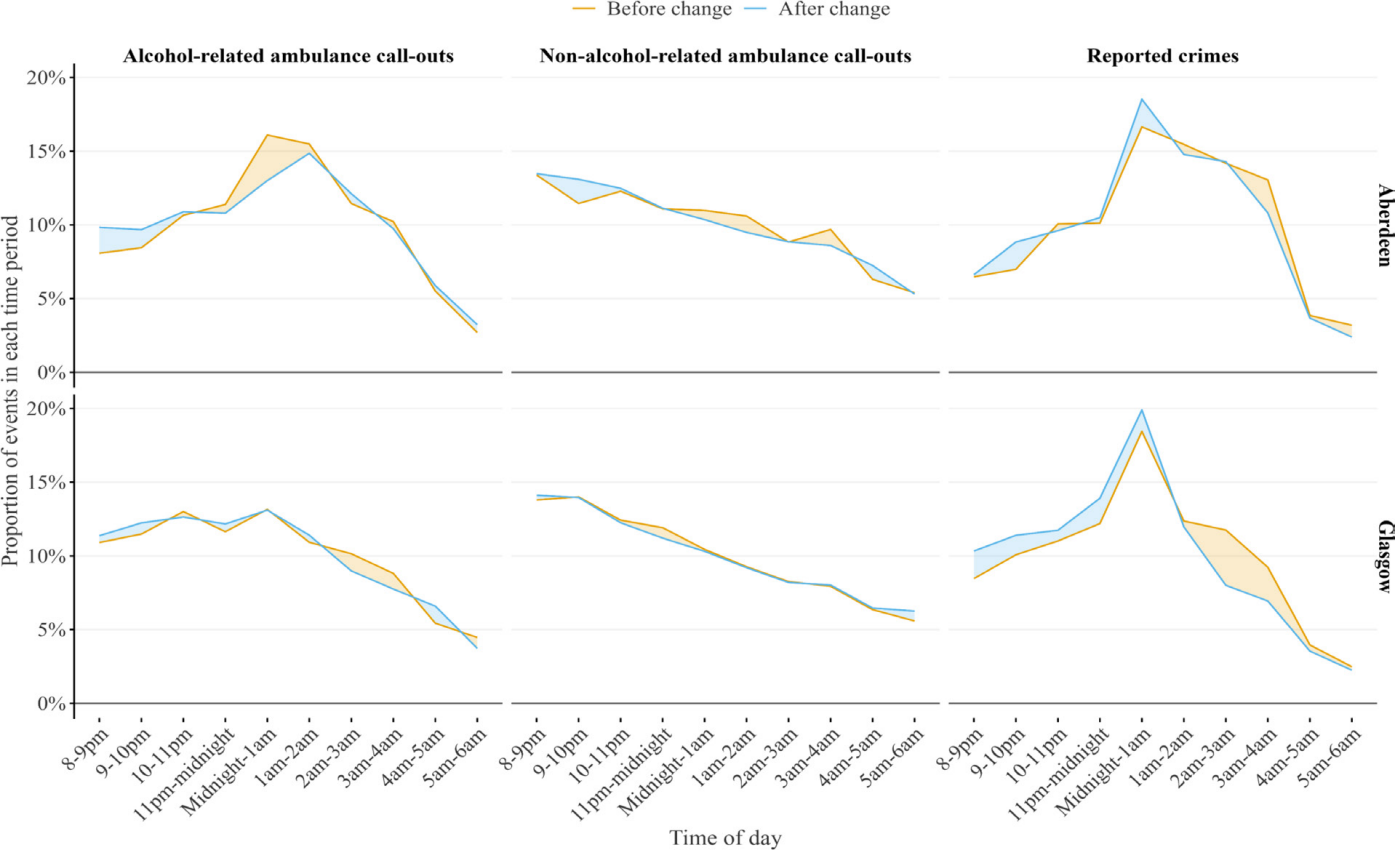
Distribution of weekend night-time alcohol-related ambulance call-out and reported crimes in Aberdeen and Glasgow.

In Aberdeen, alcohol-related ambulance call-outs peaked between 00:00 and 00:59 before the new policy but shifted to 01:00–01:59 after the policy change (figure 2); such a shift was not observed in the control city (Edinburgh) and for reported crimes (online supplemental figure A2).

The estimated policy effect for Aberdeen using a staggered policy variable when the model included only statistically significant covariates was a significant average relative increase of 4.643 extra call-outs per week in Aberdeen following the policy change compared with Edinburgh (corresponding to a 11.4% relative increase in Aberdeen compared with Edinburgh) (table 1). Increasing policy effects were consistently observed for Aberdeen across various sensitivity analyses. However, the policy effect term was not significant when using the SC method. Subgroup analyses revealed that the policy effects in Aberdeen were comparatively higher for males (coefficient=3.995, p value=0.002, 95% CI 1.487 to 6.502) and individuals under 45 years of age (coefficient=14.904, p value <0.001, 95% CI 9.983 to 19.825) compared with their respective counterparts (online supplemental tables A9–A12). Similarly, when only statistically significant covariates were included in the model, the effect was 3.442 (p value=0.035, 95% CI 0.239 to 6.645) for reported crimes in Aberdeen (table 2). This means an estimated average of 3.442 extra reported crimes per week in Aberdeen than in Edinburgh, following the policy change (an 8.5% relative increase of reported crimes for Aberdeen compared with Edinburgh). Across our sensitivity analyses, in most cases, the estimated policy effects show increases after the policy change at 5% significance level (table 2). In some cases, the estimated policy effects indicate decreases after the policy change, but these effect size estimators are not statistically significant.

**Table 1:** Effect of policy changes on weekend night-time alcohol-related ambulance call-outs in Aberdeen and Glasgow

Primary outcome, weekend night-time alcohol-related ambulance call-outs	Main analysis	Sensitivity analysis		Synthetic control*	
	Outcome: number of incidents	Outcome: number of incidents (extended time series, May 2015–July 2022)	Outcome: population adjusted incident rates	Outcome: population adjusted incident rates	
	Coefficient (95% CI); P value	Coefficient (95% CI); P value	Coefficient (95% CI); P value	Coefficient	P value
Intervention area: Aberdeen					
Staggered policy covariate					
Policy effect (all covariates)	4.641 (0.279 to 9.002); 0.037	5.824 (2.225 to 9.423); 0.002	0.211 (0.064 to 0.358); 0.005	0.095	0.222
Policy effect (significant covariates)	4.643 (0.292 to 8.994); 0.036	7.061 (3.902 to 10.220); <0.001	0.222 (0.133 to 0.312); <0.001		
Dummy policy covariate, sensitivity analysis					
Policy effect (all covariates)	2.486 (−0.483 to 5.455); 0.101	2.898 (−0.078 to 5.873); 0.056	0.117 (0.017 to 0.217); 0.022	0.161	0.185
Policy effect (significant covariates)	2.565 (−0.470 to 5.599); 0.098	2.970 (−0.014 to 5.955); 0.051	0.144 (0.081 to 0.207); <0.001		
Dummy policy covariate with policy implemented with at least half strength, sensitivity analysis					
Policy effect (all covariates)	2.018 (−1.369 to 5.406); 0.243	3.145 (0.136 to 6.154); 0.041	0.051 (−0.053 to 0.155); 0.332	–	–
Policy effect (significant covariates)	1.744 (−1.585 to 5.072); 0.305	4.473 (1.846 to 7.100); 0.001	0.054 (−0.145 to 0.254); 0.593	–	–
Intervention area: Glasgow					
Dummy policy covariate					
Policy effect (all covariates)	−2.307 (−6.942 to 2.329); 0.329	−4.016 (−8.027 to −0.005); 0.05	−0.035 (−0.108 to 0.039); 0.358	0.047	0.926
Policy effect (significant covariates)	−2.951 (−6.423 to 0.520); 0.096	−5.110 (−7.491 to −2.730); <0.001	−0.040 (−0.100 to 0.021); 0.198		

* Effect size for synthetic control adjusted by the covariates, number of on-premises alcohol outlets (population adjusted) and per capita gross disposable household income.

**Table 2:** Effect of policy changes on weekend recorded crimes in Aberdeen and Glasgow

Secondary outcome, weekend night-time recorded crimes	Main analysis	Sensitivity analysis		Synthetic control*	
	Outcome: number of incidents	Outcome: number of incidents (extended time series, May 2015–July 2022)	Outcome: population adjusted incident rates	Outcome: population adjusted incident rates	
	Coefficient (95% CI); P value	Coefficient (95% CI); P value	Coefficient (95% CI); P value	Coefficient	P value
Intervention area: Aberdeen					
Staggered policy covariate					
Policy effect (all covariates)	4.596 (−2.722 to 11.914); 0.218	5.770 (−0.514 to 12.055); 0.072	0.074 (−0.111 to 0.259); 0.434	0.013	0.444
Policy effect (significant covariates)	3.442 (0.239 to 6.645); 0.035	3.187 (0.564 to 5.810); 0.017	0.163 (0.025 to 0.301); 0.020		
Dummy policy covariate, sensitivity analysis					
Policy effect (all covariates)	−1.459 (−5.286 to 2.368); 0.455	−1.551 (−5.072 to 1.969); 0.388	−0.076 (−0.186 to 0.034); 0.174	0.024	0.407
Policy effect (significant covariates)	1.192 (−0.985 to 3.368); 0.283	0.960 (−1.269 to 3.190); 0.398	−0.075 (−0.184 to 0.034); 0.179		
Dummy policy covariate with policy implemented with at least half strength, sensitivity analysis					
Policy effect (all covariates)	3.205 (0.332 to 6.078); 0.029	4.020 (1.190 to 6.850); 0.005	0.181 (0.084 to 0.279); <0.001	–	–
Policy effect (significant covariates)	3.245 (1.128 to 5.361); 0.003	3.350 (1.304 to 5.395); 0.001	0.174 (0.079 to 0.268); <0.001	–	–
Intervention area: Glasgow					
Dummy policy covariate					
Policy effect (all covariates)	−0.229 (−3.037 to 2.579); 0.873	−4.389 (−9.877 to 1.098); 0.117	−0.041 (−0.123 to 0.041); 0.332	0.133	0.926
Policy effect (significant covariates)	−0.403 (−3.224 to 2.418); 0.779	−2.734 (−7.623 to 2.155); 0.273	0.003 (−0.030 to 0.036); 0.860		

* Effect size for synthetic control adjusted by the covariates, number of on-premises alcohol outlets (population adjusted), and per capita gross disposable household income.

In contrast, Glasgow showed no significant policy effect at 5% significance level on alcohol-related ambulance call-outs and on reported crimes based on ARIMA models (tables1  2). Moreover, Glasgow results varied substantially across the range of sensitivity analyses. Further sensitivity analyses for ambulance call-outs and reported crimes are given in online supplemental tables A6–A8 and table A13.

We also conducted falsification tests for both primary and secondary outcomes; results are provided in online supplemental tables A14–A16.

**Figure 3:**
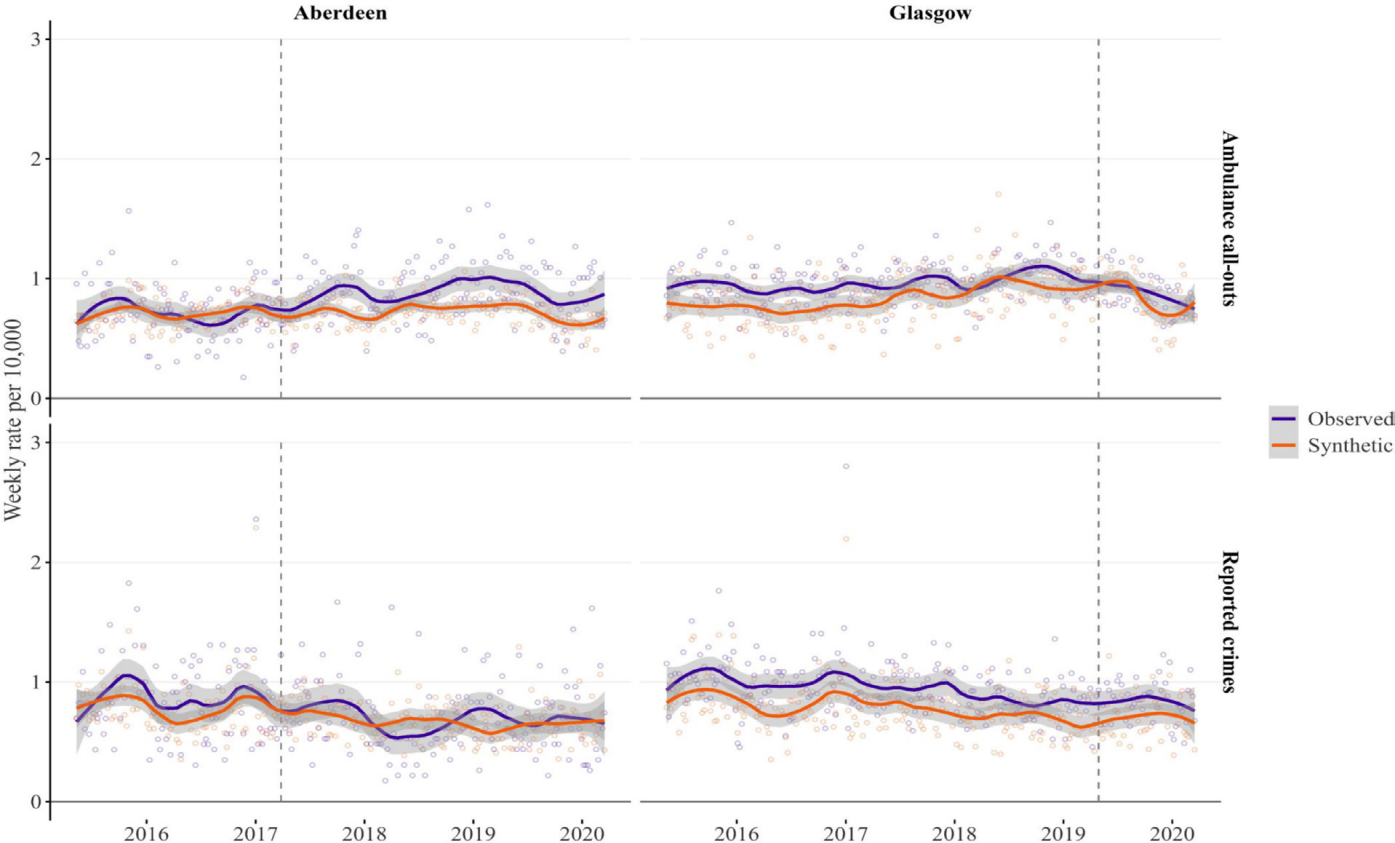
Rate of weekend night-time alcohol-related ambulance call-outs and reported crimes in Aberdeen, Glasgow and synthetic Aberdeen and synthetic Glasgow. Dots represent weekly data; solid lines indicate LOESS-smoothed trends and shaded areas represent 95% CIs; vertical dashed line represents start of intervention.

In figure 3, we compared the rates of weekend night-time alcohol-related ambulance call-outs and reported crimes in Aberdeen and Glasgow with their respective synthetic counterparts (a counterfactual scenario that assumes no policy changes occurred). For alcohol-related ambulance call-out rates, the observed and synthetic trends in Aberdeen prior to the intervention show the best fit compared with Glasgow. After the policy change, the observed rates in Aberdeen mostly lie above the synthetic rates, suggesting an increase in call-outs due to the policy. In contrast, Glasgow shows considerable overlap between observed and synthetic rates after the policy change, indicating no clear effect.

For reported crime rates, although some overlap exists between the observed and synthetic trends in Aberdeen after the policy change, the observed rates lie above the synthetic rates at most time points, suggesting a potential increase. In Glasgow, the observed crime rates also mostly lie above the synthetic rates following the policy change, implying an increase. However, the pre-intervention fit between observed and synthetic crime rates is weaker in Glasgow than in Aberdeen.

## Discussion

The results revealed clear differences in the effects of the policy changes in the two cities. In Aberdeen, the later trading hours policy applied to 38 bars and pubs led to a statistically significant 11.4% increase in alcohol-related ambulance call-outs and 8.5% increase in reported crimes relative to Edinburgh (control city) at weekend night-times. Our findings in Aberdeen align with previous studies reporting an increase of alcohol harms after the implementation of extended alcohol premises hours policies.^11 12^ Our findings also show a temporal shift in peak times for alcohol-related ambulance call-outs in Aberdeen, from 00:00–00:59 to 01:00–01:59, suggesting that the extended premises hours may have altered drinking patterns or increased alcohol availability, shifting the time at which individuals required ambulance services. Additionally, we found the impact of the policy change on alcohol-related ambulance call-outs in Aberdeen was significantly higher among men and individuals under 45 years of age. This is consistent with prior research showing that younger individuals, particularly men, are more likely to engage in risky drinking behaviours and are more at risk of alcohol-related ambulance call-outs.^7^

In contrast, no significant association was observed due to the later trading hours policy for the 10 nightclubs in Glasgow in either alcohol-related ambulance call-outs or reported crimes when compared with our control, Edinburgh. Our Glasgow findings, of no consistent significant effect, conflict with one study that found a 1-h extension to existing late-night licences was associated with a 34% more alcohol-related ambulance call-outs in Amsterdam, Netherlands.^22^ Our findings are consistent with conclusions drawn from a systematic review (albeit based on a small number of studies), which suggested that extending alcohol premises hours by two or more hours is significantly associated with increased alcohol-related harms, while mixed results were found for extensions of less than 2 h.^14^

A key strength of our study is its robust design, which incorporated a structured prespecified control selection process.^19^ We triangulated our findings across modelling approaches to enhance reliability of the results. Unlike previous research that often overlooks staggered policy implementation, venue capacity and premises type,^16^ our design incorporated a staggered policy covariate for Aberdeen based on the person-hours, calculated based on the number of permitted premises, their maximum capacities and extended hours.

While the study employs robust methodological approaches including CITS and SC methods, several limitations should be acknowledged. Although such a design strengthens causal inference relative to simple observational comparisons, they rely on assumptions that cannot be fully verified in practice, including the parallel trends assumption and the potential for unmeasured time-varying confounding factors.^23 24^ Violations of these assumptions may bias estimated effects. In addition, crime outcomes may be affected by under-reporting and administrative data may be subject to measurement error or changes in reporting practices over time. Hence, while our design is intended to estimate causal effects, findings should be interpreted in light of these methodological constraints. We used the approval dates rather than implementation dates, since it is unclear whether all approved premises actually implemented extended hours; qualitative findings indicate that use of the extended hours was unpredictable across both cities due to mixed demand (paper forthcoming).

The primary CITS analysis was based on counts rather than rates because the population denominators for the relevant at-risk population (ie, individuals present in licensed premises during late-night hours) are not directly observable and are likely to vary over time due to tourism, student populations and mobility patterns. As a result, rate-based analyses based on cities’ population as denominators may introduce measurement error that could bias estimates. We therefore specified CITS models based on counts as the main analysis, while presenting rate-based CITS analyses as complementary sensitivity analyses to assess the robustness of findings to population standardisation. Population data are recorded annually; therefore, we used extrapolation technique to derive weekly population estimates. Both ARIMA and SC analyses showed similar effect direction in Aberdeen, but SC estimates were not statistically significant. This is maybe due to the frequentist (frequency based) nature of SC p values, which are limited by number of control units in the donor pool.^25^ Moreover, effect sizes are not directly comparable across models’ estimating counts versus rates, since ARIMA estimators are sensitive to scale/unit of analysis.^24^

Our findings have important policy implications, illustrating further limitations of UK licensing systems.^26^ If the volume and type of premises with later alcohol trading hours are important factors dictating harms, then policy makers should have powers to control these factors. The current system in Scotland (and in England and Wales) does not distinguish between premises types.^27^ Glasgow was only able to establish different standard trading hours for bars and nightclubs by including a robust definition of a nightclub in their licensing policy statement.^28^ Furthermore, local authorities cannot regulate the volume of premises eligible for later trading hours because each application has to be considered on its individual merits.^29^ Although we studied the impact of the 4am extension in Glasgow’s pilot with 10 nightclubs, our findings suggest that granting the same hours to additional nightclubs would carry a higher risk of negative outcomes, yet there is little to prevent multiple premises now being included. To protect public health and safety late at night, licensing systems may need to include powers to create type-specific caps on venue numbers with very late hours, for example, per population or locality, similar to practice in several North American jurisdictions.

## Conclusion

This study demonstrates the value of robust, multicomponent analysis of outcomes when assessing the impact of changes to licensing hours. While our findings confirm the general principle that widespread extending alcohol trading hours, especially late into the night, are associated with some increased harms, they also show that the intensity of these impacts is likely mediated by type and volume of outlets. It is therefore important that licensing systems can account for these factors.

## Supplementary material

10.1136/bmjph-2025-003722 online supplemental file 1

## Data Availability

Data may be obtained from a third party and are not publicly available.
