# Supplementary material for: Impact of later trading hours for bars and clubs on alcohol-related ambulance call-outs and crimes in Scotland: a controlled interrupted time series study
Source: BMJ Public Health. 2026 Apr 27;4(2):e003722. doi: 10.1136/bmjph-2025-003722 (PMC13141207; doi:10.1136/bmjph-2025-003722)
Supplement: online supplemental file 1 [file bmjph-4-2-s001.docx]

**Appendix**

**Contents**

[**Appendix 1: Control selection process and study design** 2](#_Toc221191148)

[**Appendix 1.1. Alcohol-related ambulance call-outs** 3](#_Toc221191149)

[**Appendix 1.2. Reported crimes** 5](#_Toc221191150)

[**Appendix 2: Analysis** 9](#_Toc221191151)

[**Appendix 2.1. Descriptive analysis** 9](#_Toc221191152)

[**Appendix 2.2. Description of confounding variables** 15](#_Toc221191153)

[***Appendix 2.2.1. Data sources for confounding variables*** 15](#_Toc221191154)

[**Appendix 2.3. Secondary and Sub-group analysis** 16](#_Toc221191155)

[**Appendix 2.4: Falsification test** 24](#_Toc221191156)

[**Appendix 3. Synthetic control** 28](#_Toc221191157)

[**Appendix 3.1. Alcohol-related ambulance call-outs (Aberdeen)** 28](#_Toc221191158)

[***Appendix 3.1.1. Model specification and validation test*** 28](#_Toc221191159)

[***Appendix 3.1.2. Main analysis and sensitivity tests*** 29](#_Toc221191160)

[**Appendix 3.2. Alcohol-related ambulance call-outs (Glasgow)** 35](#_Toc221191161)

[***Appendix 3.2.1. Model specification and validation test*** 35](#_Toc221191162)

[***Appendix 3.2.2. Main analysis and sensitivity tests*** 35](#_Toc221191163)

[**Appendix 3.3. Reported crimes (Aberdeen)** 41](#_Toc221191164)

[***Appendix 3.3.1. Model specification and validation test*** 41](#_Toc221191165)

[***Appendix 3.3.2. Main analysis and sensitivity tests*** 41](#_Toc221191166)

[**Appendix 3.4. Reported crimes (Glasgow)** 48](#_Toc221191167)

[***Appendix 3.4.1. Model specification and validation test*** 48](#_Toc221191168)

[***Appendix 3.4.2. Main analysis and sensitivity tests*** 48](#_Toc221191169)

[**Appendix 4. Synthetic control and ARIMA** 55](#_Toc221191170)

# **Appendix 1: Control selection process and study design**

**
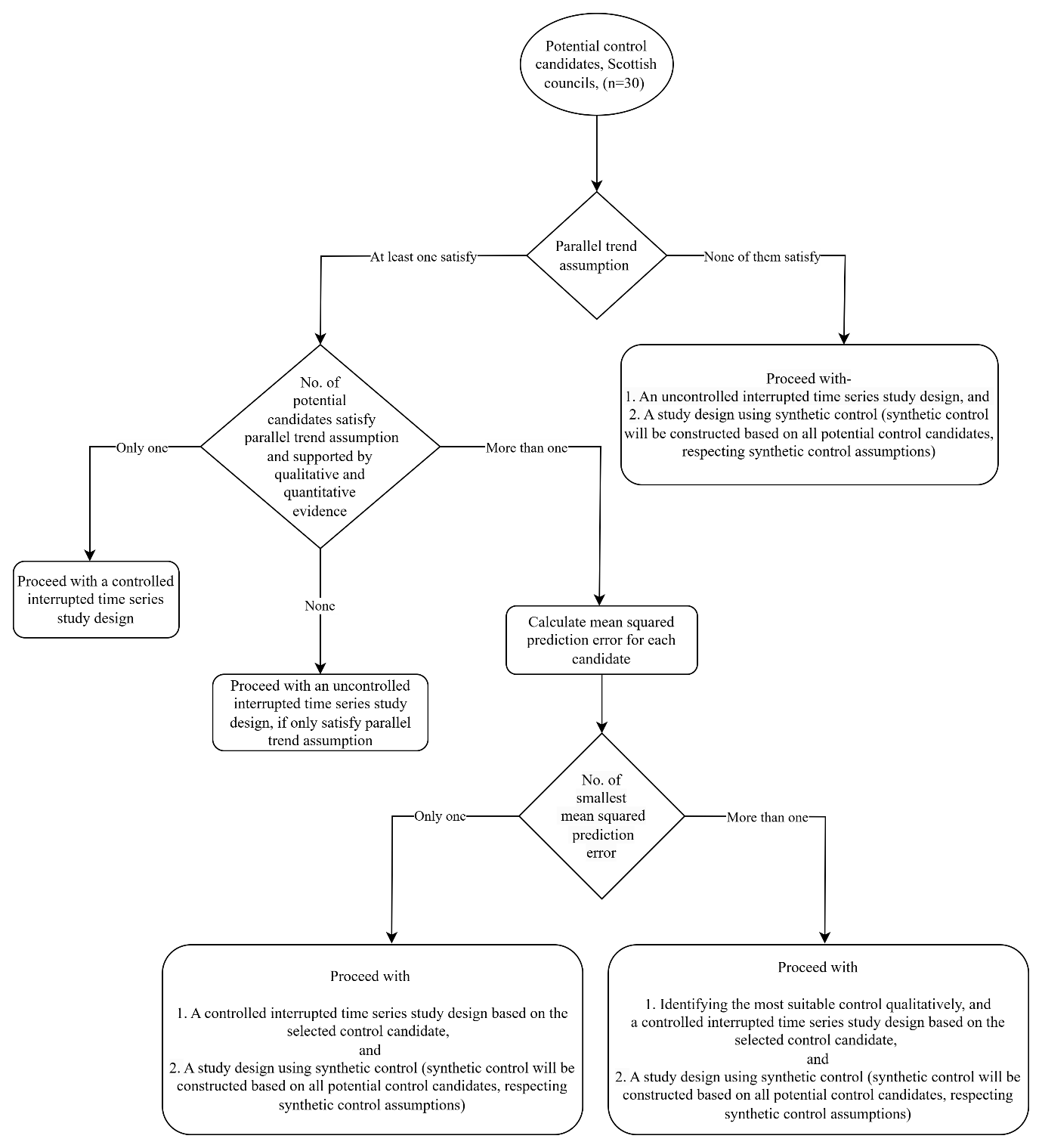
**

**Figure A1: Flow diagram for the control selection procedure**

## **Appendix 1.1. Alcohol-related ambulance call-outs**

In step 1, we tested the parallel trend assumption both graphically and statistically for 30 potential Scottish cities/council areas, excluding Aberdeen and Glasgow. We estimated the slope coefficients for Aberdeen and Glasgow against each of the 30 potential control candidates, using data from the pre-intervention periods only. Any control candidate with a statistically significant slope coefficient, indicating a statistically significant difference in trends from Aberdeen or Glasgow, was excluded. As a result, Stirling was excluded as a control for Aberdeen, while Angus, Argyll and Bute, Dundee, East Ayrshire, Highland, Perth and Kinross, Scottish Borders, and Stirling were excluded as controls for Glasgow.

In step 2, we reviewed the licensing policies of the remaining potential control cities/councils and excluded those with similar extended premises hours policies to Aberdeen or Glasgow. Additionally, we removed candidates whose population-adjusted density of on-premises alcohol outlets was more than one standard deviation above or below the densities of the intervention areas (Aberdeen and Glasgow).

In step 3, we computed the mean squared prediction errors (MSPEs) for all remaining control candidates. The MSPE represents the mean squared differences between the intervention areas (Aberdeen/Glasgow) and the potential control candidates during the pre-intervention periods. For Aberdeen, the candidates with the lowest MSPEs were Edinburgh, Fife, and North Lanarkshire, while for Glasgow, Edinburgh had the lowest MSPE. Ultimately, Edinburgh was chosen as the control for both Aberdeen and Glasgow. For Aberdeen, this decision was informed by Edinburgh’s significant contribution in synthetic control analysis compared to Fife and North Lanarkshire, alongside our a priori decision to use Edinburgh as a control.

**Step 1: Testing parallel trend assumptions graphically and statistically**

| **Parallel trend assumption test: Ambulance call-outs (based on rate)** | | | | | | |
| --- | --- | --- | --- | --- | --- | --- |
| **Council name** | **Aberdeen** | | | **Glasgow** | | |
|  | **Slope** | **P-value** | **Slope** | | **P-value** |  |
| Aberdeenshire | -0·0009 | 0·268 | -0·0001 | | 0·763 |  |
| Angus | -0·0002 | 0·826 | -0·0006 | | 0·049 |  |
| Argyll and Bute | -0·0022 | 0·054 | -0·0016 | | <0·001 |  |
| City of Edinburgh | -0·0017 | 0·061 | 0·0003 | | 0·248 |  |
| Clackmannanshire | -0·0009 | 0·554 | -0·0006 | | 0·240 |  |
| Dumfries and Galloway | -0·0007 | 0·468 | -0·0004 | | 0·191 |  |
| Dundee City | -0·0007 | 0·514 | -0·0009 | | 0·010 |  |
| East Ayrshire | -0·0014 | 0·169 | -0·0009 | | 0·010 |  |
| East Dunbartonshire | -0·0011 | 0·258 | 0·0004 | | 0·097 |  |
| East Lothian | 0·0003 | 0·760 | 0·0000 | | 0·913 |  |
| East Renfrewshire | -0·0019 | 0·050 | -0·0002 | | 0·384 |  |
| Falkirk | 0·0003 | 0·803 | -0·0003 | | 0·319 |  |
| Fife | -0·0007 | 0·389 | -0·0003 | | 0·186 |  |
| Highland | 0·0006 | 0·469 | -0·0006 | | 0·007 |  |
| Inverclyde | 0·0013 | 0·321 | 0·0005 | | 0·219 |  |
| Midlothian | -0·0005 | 0·639 | -0·0001 | | 0·847 |  |
| Moray | -0·0006 | 0·612 | -0·0007 | | 0·055 |  |
| North Ayrshire | 0·0004 | 0·757 | 0·0004 | | 0·221 |  |
| North Lanarkshire | 0·0000 | 0·989 | 0·0003 | | 0·286 |  |
| Orkney Islands | 0·0003 | 0·825 | 0·0005 | | 0·307 |  |
| Perth and Kinross | -0·0014 | 0·169 | 0·0007 | | 0·009 |  |
| Renfrewshire | 0·0001 | 0·925 | 0·0003 | | 0·325 |  |
| Scottish Borders | -0·0018 | 0·095 | 0·0012 | | 0·001 |  |
| Shetland Islands | -0·0008 | 0·587 | -0·0002 | | 0·718 |  |
| South Ayrshire | -0·0005 | 0·688 | -0·0004 | | 0·186 |  |
| South Lanarkshire | -0·0013 | 0·195 | -0·0002 | | 0·536 |  |
| Stirling | -0·0026 | 0·019 | -0·0008 | | 0·017 |  |
| West Dunbartonshire | 0·0014 | 0·246 | -0·0003 | | 0·477 |  |
| West Lothian | 0·0001 | 0·912 | -0·0001 | | 0·620 |  |
| Western Isles - Eilean Siar | -0·0001 | 0·945 | -0·0008 | | 0·090 |  |

**Step 2: Assess qualitatively and quantitatively the councils/cities that passed parallel test assumption at Step 1**

| **Potential control candidates that satisfied parallel trend assumption** | | | | | |
| --- | --- | --- | --- | --- | --- |
| **Aberdeen (no. of on sale licenses, 432; density per 1000 population, 1·90 (SD 0·87))** | | | **Glasgow (no. of on sale licenses, 1352; density per 1000 population, 2·13 (SD 0·85))** | | |
| **Council name** | **Number of on sale licenses, 2022** | **Density per 1000 population** | **Council name** | **Number of on sale licenses, 2022** | **Density per 1000 population** |
| Aberdeenshire | 370 | 1·40 | Aberdeenshire** | 370 | 1·40 |
| Angus | 246 | 2·12 | City of Edinburgh | 1467 | 2·75 |
| Argyll and Bute** | 451 | 5·35 | Clackmannanshire* | 76 | 1·48 |
| City of Edinburgh | 1467 | 2·75 | Dumfries and Galloway** | 456 | 3·10 |
| Clackmannanshire* | 76 | 1·48 | East Dunbartonshire* | 119 | 1·08 |
| Dumfries and Galloway** | 456 | 3·10 | East Lothian | 213 | 1·95 |
| Dundee City | 307 | 2·07 | East Renfrewshire** | 118 | 1·21 |
| East Ayrshire | 181 | 1·49 | Falkirk** | 199 | 1·22 |
| East Dunbartonshire* | 119 | 1·08 | Fife | 681 | 1·83 |
| East Lothian | 213 | 1·95 | Inverclyde | 125 | 1·64 |
| East Renfrewshire | 118 | 1·21 | Midlothian | 132 | 1·37 |
| Falkirk | 199 | 1·22 | Moray | 262 | 2·74 |
| Fife | 681 | 1·83 | North Ayrshire | 255 | 1·91 |
| Highland** | 922 | 3·90 | North Lanarkshire** | 394 | 1·16 |
| Inverclyde | 125 | 1·64 | Orkney Islands | 66 | 2·96 |
| Midlothian | 132 | 1·37 | Renfrewshire | 279 | 1·55 |
| Moray | 262 | 2·74 | Shetland Islands** | 104 | 4·53 |
| North Ayrshire | 255 | 1·91 | South Ayrshire | 271 | 2·42 |
| North Lanarkshire | 394 | 1·16 | South Lanarkshire* | 457 | 1·42 |
| Orkney Islands** | 66 | 2·96 | West Dunbartonshire | 140 | 1·58 |
| Perth and Kinross** | 436 | 2·86 | West Lothian** | 211 | 1·13 |
| Renfrewshire | 279 | 1·55 | Western Isles - Eilean Siar | 67 | 2·56 |
| Scottish Borders** | 343 | 2·96 |  |  |  |
| Shetland Islands** | 104 | 4·53 |  |  |  |
| South Ayrshire | 271 | 2·42 |  |  |  |
| South Lanarkshire* | 457 | 1·42 |  |  |  |
| West Dunbartonshire | 140 | 1·58 |  |  |  |
| West Lothian | 211 | 1·13 |  |  |  |
| Western Isles - Eilean Siar | 67 | 2·56 |  |  |  |
| *Excluded due to extension of alcohol premises hours | | | |  |  |
| **Excluded if density of on-sale premises, ± 1 SD that of Aberdeen and Glasgow | | | |  |  |

**Step 3: Calculate mean square prediction error based on pre-intervention data to choose control candidate based on lowest value**

| **Potential control candidates satisfied parallel trend assumption and passed qualitative and quantitative assessment** | | | | |
| --- | --- | --- | --- | --- |
| **Intervention: Aberdeen** | | **Intervention: Glasgow** | |  |
| **Council name** | **Mean squared prediction error (MSPE)** | **Council name** | **Mean squared prediction error (MSPE)** |  |
| Aberdeenshire | 0·23 | City of Edinburgh | 0·07 |  |
| Angus | 0·14 | East Lothian | 0·29 |  |
| City of Edinburgh | 0·07 | Fife | 0·13 |  |
| Dundee City | 0·10 | Inverclyde | 0·16 |  |
| East Ayrshire | 0·11 | Midlothian | 0·20 |  |
| East Lothian | 0·16 | Moray | 0·24 |  |
| East Renfrewshire | 0·28 | North Ayrshire | 0·11 |  |
| Falkirk | 0·11 | Orkney Islands | 0·63 |  |
| Fife | 0·07 | Renfrewshire | 0·13 |  |
| Inverclyde | 0·14 | South Ayrshire | 0·16 |  |
| Midlothian | 0·12 | West Dunbartonshire | 0·16 |  |
| Moray | 0·16 | Western Isles - Eilean Siar | 0·45 |  |
| North Ayrshire | 0·12 |  |  |  |
| North Lanarkshire | 0·07 |  |  |  |
| Renfrewshire | 0·08 |  |  |  |
| South Ayrshire | 0·13 |  |  |  |
| West Dunbartonshire | 0·12 |  |  |  |
| West Lothian | 0·12 |  |  |  |
| Western Isles - Eilean Siar | 0·31 |  |  |  |

## **Appendix 1.2. Reported crimes**

In step 1, for reported crimes, Shetland Islands was excluded as a control for Aberdeen, while Aberdeenshire, East Dunbartonshire, East Lothian, East Renfrewshire, Highland, North Lanarkshire, South Ayrshire, and South Lanarkshire were excluded as controls for Glasgow.

In step 2, Argyll and Bute, Clackmannanshire, Dumfries and Galloway, East Dunbartonshire, Highland, Orkney Islands, Perth and Kinross, Scottish Borders, South Lanarkshire, and Stirling excluded as controls for Aberdeen. Similarly, for Glasgow, Argyll and Bute, Clackmannanshire, Dumfries and Galloway, Falkirk, Orkney Islands, Perth and Kinross, Scottish Borders, Shetland Islands, and West Lothian were excluded.

In step 3, Edinburgh was chosen as the control for both Aberdeen and Glasgow for reported crimes based on the lowest MSPEs among the surviving control candidates.

**Step 1: Testing parallel trend assumptions graphically and statistically**

| **Parallel trend assumption test: Police call-outs (based on rate)** | | | | |
| --- | --- | --- | --- | --- |
| **Council name** | **Aberdeen** | | **Glasgow** | |
|  | **Slope** | **P-value** | **Slope** | **P-value** |
| Aberdeenshire | 0·0000 | 0·973 | -0·0008 | 0·012 |
| Angus | -0·0015 | 0·289 | -0·0003 | 0·495 |
| Argyll and Bute | -0·0003 | 0·866 | -0·0009 | 0·078 |
| City of Edinburgh | 0·0006 | 0·558 | -0·0002 | 0·538 |
| Clackmannanshire | 0·0007 | 0·665 | -0·0002 | 0·695 |
| Dumfries and Galloway | 0·0004 | 0·757 | -0·0006 | 0·163 |
| Dundee City | -0·0002 | 0·899 | 0·0001 | 0·772 |
| East Ayrshire | -0·0019 | 0·168 | 0·0001 | 0·851 |
| East Dunbartonshire | -0·0008 | 0·514 | -0·0011 | 0·001 |
| East Lothian | -0·0001 | 0·924 | -0·0011 | 0·007 |
| East Renfrewshire | -0·0010 | 0·461 | -0·0007 | 0·034 |
| Falkirk | 0·0012 | 0·414 | -0·0007 | 0·061 |
| Fife | 0·0005 | 0·643 | -0·0002 | 0·505 |
| Highland | 0·0024 | 0·081 | 0·0007 | 0·042 |
| Inverclyde | -0·0002 | 0·911 | -0·0009 | 0·070 |
| Midlothian | 0·0011 | 0·492 | -0·0005 | 0·210 |
| Moray | 0·0003 | 0·837 | -0·0005 | 0·305 |
| North Ayrshire | 0·0008 | 0·512 | 0·0003 | 0·428 |
| North Lanarkshire | -0·0006 | 0·562 | -0·0009 | 0·001 |
| Orkney Islands | 0·0024 | 0·369 | -0·0007 | 0·349 |
| Perth and Kinross | 0·0002 | 0·878 | -0·0005 | 0·118 |
| Renfrewshire | 0·0002 | 0·834 | -0·0007 | 0·079 |
| Scottish Borders | 0·0004 | 0·758 | -0·0006 | 0·075 |
| Shetland Islands | 0·0067 | 0·009 | 0·0012 | 0·101 |
| South Ayrshire | 0·0009 | 0·569 | -0·0008 | 0·035 |
| South Lanarkshire | 0·0000 | 0·971 | -0·0008 | 0·005 |
| Stirling | 0·0012 | 0·433 | -0·0001 | 0·809 |
| West Dunbartonshire | 0·0022 | 0·207 | -0·0002 | 0·687 |
| West Lothian | 0·0002 | 0·868 | -0·0005 | 0·106 |
| Western Isles - Eilean Siar | 0·0036 | 0·063 | -0·0006 | 0·325 |

**Step 2: Assess qualitatively and quantitatively to the councils/cities passed parallel test assumption**

| **Potential control candidates satisfied parallel trend assumption** | | | | | | |
| --- | --- | --- | --- | --- | --- | --- |
| **Aberdeen (no. of on sale licenses, 432; density per 1000 population, 1·90 (SD 0·87))** | | | | **Glasgow (no. of on sale licenses, 1352; density per 1000 population, 2·13 (SD 0·85))** | | |
| **Council name** | **Number of on sale licenses, 2022** | **Council name** | **Number of on sale licenses, 2022** | | **Council name** | **Number of on sale licenses, 2022** |
| Aberdeenshire | 370 | 1·40 | Angus | | 246 | 2·12 |
| Angus | 246 | 2·12 | Argyll and Bute** | | 451 | 5·35 |
| Argyll and Bute** | 451 | 5·35 | City of Edinburgh | | 1467 | 2·75 |
| City of Edinburgh | 1467 | 2·75 | Clackmannanshire* | | 76 | 1·48 |
| Clackmannanshire* | 76 | 1·48 | Dumfries and Galloway** | | 456 | 3·10 |
| Dumfries and Galloway** | 456 | 3·10 | Dundee City | | 307 | 2·07 |
| Dundee City | 307 | 2·07 | East Ayrshire | | 181 | 1·49 |
| East Ayrshire | 181 | 1·49 | Falkirk** | | 199 | 1·22 |
| East Dunbartonshire* | 119 | 1·08 | Fife | | 681 | 1·83 |
| East Lothian | 213 | 1·95 | Inverclyde | | 125 | 1·64 |
| East Renfrewshire | 118 | 1·21 | Midlothian | | 132 | 1·37 |
| Falkirk | 199 | 1·22 | Moray | | 262 | 2·74 |
| Fife | 681 | 1·83 | North Ayrshire | | 255 | 1·91 |
| Highland** | 922 | 3·90 | Orkney Islands** | | 66 | 2·96 |
| Inverclyde | 125 | 1·64 | Perth and Kinross* | | 436 | 2·86 |
| Midlothian | 132 | 1·37 | Renfrewshire | | 279 | 1·55 |
| Moray | 262 | 2·74 | Scottish Borders** | | 343 | 2·96 |
| North Ayrshire | 255 | 1·91 | Shetland Islands** | | 104 | 4·53 |
| North Lanarkshire | 394 | 1·16 | Stirling | | 273 | 2·84 |
| Orkney Islands** | 66 | 2·96 | West Dunbartonshire | | 140 | 1·58 |
| Perth and Kinross* | 436 | 2·86 | West Lothian** | | 211 | 1·13 |
| Renfrewshire | 279 | 1·55 | Western Isles - Eilean Siar | | 67 | 2·56 |
| Scottish Borders** | 343 | 2·96 |  | |  |  |
| South Ayrshire | 271 | 2·42 |  | |  |  |
| South Lanarkshire* | 457 | 1·42 |  | |  |  |
| Stirling** | 273 | 2·84 |  | |  |  |
| West Dunbartonshire | 140 | 1·58 |  | |  |  |
| West Lothian | 211 | 1·13 |  | |  |  |
| Western Isles - Eilean Siar | 67 | 2·56 |  | |  |  |
| *Excluded due to extension of alcohol premises hours | | | | | | |
| **Excluded if density of on-sale premises, ± 1 SD that of Aberdeen and Glasgow | | | | | | |

**Step 3: Calculate mean square prediction error based on pre-intervention data to choose control candidate based on lowest value.**

| **Potential control candidates satisfied parallel trend assumption and passed qualitative and quantitative assessment** | | | | |
| --- | --- | --- | --- | --- |
| **Council name** | **Mean squared prediction error (MSPE)** | **Council name** | **Mean squared prediction error (MSPE)** |  |
| Aberdeenshire | 0·26 | Angus | 0·23 |  |
| Angus | 0·19 | City of Edinburgh | 0·09 |  |
| City of Edinburgh | 0·09 | Dundee City | 0·12 |  |
| Dundee City | 0·15 | East Ayrshire | 0·25 |  |
| East Ayrshire | 0·19 | Fife | 0·16 |  |
| East Lothian | 0·37 | Inverclyde | 0·34 |  |
| East Renfrewshire | 0·50 | Midlothian | 0·28 |  |
| Falkirk | 0·18 | Moray | 0·24 |  |
| Fife | 0·13 | North Ayrshire | 0·19 |  |
| Inverclyde | 0·31 | Orkney Islands | 0·63 |  |
| Midlothian | 0·26 | Renfrewshire | 0·22 |  |
| Moray | 0·20 | Scottish Borders | 0·36 |  |
| North Ayrshire | 0·15 | Stirling | 0·32 |  |
| North Lanarkshire | 0·15 | West Dunbartonshire | 0·23 |  |
| Renfrewshire | 0·16 | Western Isles - Eilean Siar | 0·67 |  |
| South Ayrshire | 0·26 |  |  |  |
| West Dunbartonshire | 0·27 |  |  |  |
| West Lothian | 0·13 |  |  |  |
| Western Isles - Eilean Siar | 0·57 |  |  |  |

# **Appendix 2: Analysis**

## **Appendix 2.1. Descriptive analysis**

| **Table A1. Total number of weekend night-time alcohol-related ambulance call-outs and reported crimes by Scottish cities from 1 May 2015 to 20 March 2020** | | |
| --- | --- | --- |
| **Scottish cities** | **Alcohol-related ambulance call-outs** | **Recorded crimes** |
| Aberdeen City | 4,765 | 4,425 |
| Aberdeenshire | 2,350 | 2,918 |
| Angus | 1,693 | 1,806 |
| Argyll & Bute | 1,309 | 1,310 |
| City of Edinburgh | 10,407 | 9,354 |
| Clackmannanshire | 999 | 854 |
| Dumfries & Galloway | 2,217 | 2,646 |
| Dundee City | 3,256 | 3,374 |
| East Ayrshire | 1,884 | 1,689 |
| East Dunbartonshire | 969 | 792 |
| East Lothian | 1,314 | 1,003 |
| East Renfrewshire | 744 | 580 |
| Eilean Siar | 305 | 237 |
| Falkirk | 2,729 | 2,781 |
| Fife | 6,341 | 5,795 |
| Glasgow City | 15,024 | 14,734 |
| Highland | 3,467 | 3,605 |
| Inverclyde | 1,433 | 1,044 |
| Midlothian | 1,440 | 1,295 |
| Moray | 1,384 | 1,519 |
| North Ayrshire | 2,792 | 2,156 |
| North Lanarkshire | 6,053 | 5,021 |
| Orkney Islands | 152 | 248 |
| Perth & Kinross | 2,081 | 1,581 |
| Renfrewshire | 3,088 | 2,663 |
| Scottish Borders | 1,820 | 1,315 |
| Shetland Islands | 249 | 267 |
| South Ayrshire | 2,015 | 1,602 |
| South Lanarkshire | 5,034 | 3,962 |
| Stirling | 1,459 | 1,191 |
| West Dunbartonshire | 1,661 | 1,343 |
| West Lothian | 2,942 | 2,625 |
| **Total** | **93,376** | **85,735** |

| **Table A2. Age, sex and socio-economic deprivation* distribution of patients in alcohol-related ambulance call-outs at the weekend night-time** | | | |
| --- | --- | --- | --- |
| **Patient characteristics** | **Aberdeen, n(%)** | **Glasgow, n(%)** | **Edinburgh, n(%)** |
| **Age group (years)** |  |  |  |
| *< 25* | 1,558 (34.20) | 5,688 (39.35) | 3,436 (34.39) |
| *25-34* | 170 (3.73) | 532 (3.68) | 348 (3.48) |
| *35-44* | 866 (19.01) | 2,668 (18.46) | 1,865 (18.67) |
| *45+* | 633 (13.90) | 2,028 (14.03) | 1,263 (12.64) |
| *Unknown* | 1,328 (29.15) | 3,540 (24.49) | 3,079 (30.82) |
| **Gender** |  |  |  |
| *Female* | 1,944 (40.80) | 5,489 (36.53) | 4,264 (40.97) |
| *Male* | 2,703 (56.73) | 9,084 (60.46) | 5,856 (56.27) |
| *Others* | - | 3 (0.02) | 3 (0.03) |
| *Unknown* | 118 (2.48) | 448 (2.98) | 284 (2.73) |
| **Socio-economic deprivation** |  |  |  |
| *1 (most deprived)* | 74 (1.55) | 5,549 (36.94) | 896 (8.61) |
| *2* | 394 (8.27) | 2,063 (13.73) | 908 (8.72) |
| *3* | 749 (15.72) | 1,063 (7.08) | 921 (8.85) |
| *4* | 356 (7.47) | 960 (6.39) | 1,594 (15.32) |
| *5* | 1,174 (24.64) | 1,123 (7.48) | 1,243 (11.94) |
| *6* | 376 (7.89) | 2,106 (14.02) | 1,186 (11.40) |
| *7* | 530 (11.12) | 361 (2.40) | 557 (5.35) |
| *8* | 322 (6.76) | 1,167 (7.77) | 1,168 (11.22) |
| *9* | 298 (6.25) | 467 (3.11) | 548 (5.27) |
| *10 (less deprived)* | 492 (10.33) | 164 (1.09) | 1,386 (13.32) |
| **Total** | **4,765** | **15,024** | **10,407** |

***Socio-economic deprivation score is based on the postcode of the incident location**

| **Table A3. Summary of weekly weekend night-time alcohol-related ambulance callouts by cities** | | | | | | | | | |
| --- | --- | --- | --- | --- | --- | --- | --- | --- | --- |
| **City** | | **Year** | **Minimum** | **Maximum** | **Mean** | **SD** | **Median** | **IQR** | **Total** |
| **Interventions** | **Aberdeen** | 2015 | 10 | 36 | 18 | 5 | 18 | 7 | 636 |
|  |  | 2016 | 4 | 26 | 15 | 5 | 16 | 7 | 800 |
|  |  | 2017 | 9 | 32 | 19 | 5 | 19 | 8 | 997 |
|  |  | 2018 | 9 | 36 | 20 | 5 | 20 | 6 | 1,042 |
|  |  | 2019 | 9 | 37 | 21 | 6 | 20 | 8 | 1,066 |
|  |  | 2020 | 3 | 26 | 19 | 7 | 21 | 9 | 224 |
|  |  | **Total** | **3** | **37** | **19** | **6** | **19** | **7** | **4,765** |
|  | **Glasgow** | 2015 | 42 | 89 | 59 | 9 | 58 | 12 | 2,069 |
|  |  | 2016 | 33 | 78 | 55 | 10 | 56 | 16 | 2,878 |
|  |  | 2017 | 35 | 85 | 60 | 10 | 60 | 13 | 3,143 |
|  |  | 2018 | 37 | 92 | 64 | 10 | 63 | 12 | 3,312 |
|  |  | 2019 | 38 | 78 | 59 | 9 | 58 | 13 | 3,063 |
|  |  | 2020 | 13 | 60 | 47 | 12 | 48 | 11 | 559 |
|  |  | **Total** | **13** | **92** | **59** | **10** | **59** | **14** | **15,024** |
| **Control** | **Edinburgh** | 2015 | 25 | 61 | 40 | 10 | 38 | 16 | 1,413 |
|  |  | 2016 | 24 | 63 | 41 | 9 | 38 | 12 | 2,115 |
|  |  | 2017 | 27 | 57 | 41 | 7 | 40 | 10 | 2,124 |
|  |  | 2018 | 26 | 70 | 42 | 8 | 41 | 9 | 2,207 |
|  |  | 2019 | 20 | 59 | 41 | 8 | 42 | 9 | 2,151 |
|  |  | 2020 | 6 | 54 | 33 | 12 | 36 | 11 | 397 |
|  |  | **Total** | **6** | **70** | **41** | **9** | **40** | **11** | **10,407** |

| **Table A4. Total number of weekly weekend night-time recorded crimes according to crime types from 1 May 2015 to 20 March 2020** | | | |
| --- | --- | --- | --- |
| **Type of recorded crimes** | **Aberdeen, n(%)** | **Glasgow, n(%)** | **Edinburgh, n(%)** |
| Common Assault | 3,143 (71.03) | 10,135 (68.79) | 7,320 (78.26) |
| Common assault of an emergency worker | 505 (11.41) | 2,056 (13.95) | 751 (8.03) |
| Consuming outwith permitted hours | - | - | 4 (0.04) |
| Culpable and reckless, causing injury | 2 (0.05) | 11 (0.07) | 5 (0.05) |
| Disorderly on licensed premises | 90 (2.03) | 182 (1.24) | 55 (0.59) |
| Drunk and Incapable | 5 (0.11) | 265 (1.80) | 87 (0.93) |
| Drunk and attempting to enter licensed premises | 21 (0.47) | 23 (0.16) | 5 (0.05) |
| Drunk in charge of a child | 2 (0.05) | 18 (0.12) | 4 (0.04) |
| Drunk in or attempting to enter designated sports ground | 1 (0.02) | 2 (0.01) | 0 (0.00) |
| Licensed person, employee or agent drunk in licensed premise | 1 (0.02) | 2 (0.01) | 5 (0.05) |
| Licensed persons, other offences | 2 (0.05) | 6 (0.04) | 5 (0.05) |
| Liquor licensing laws, other offences | - | 7 (0.05) | 0 (0.00) |
| Person under 18 buying excisable liquor in bar | - | - | 2 (0.02) |
| Permitting riotous behaviour in licen.. | - | 5 (0.03) | 0 (0.00) |
| Purchasing excise liquor for consumption by person under 18 | 1 (0.02) | - | 0 (0.00) |
| Refusing to quit a licensed premise | 186 (4.20) | 294 (2.00) | 185 (1.98) |
| Robbery | 88 (1.99) | 317 (2.15) | 219 (2.34) |
| Sale of drink to person under 18 | 0 (0.00) | 6 (0.04) | 0 (0.00) |
| Serious Assault | 361 (8.16) | 1,374 (9.33) | 677 (7.24) |
| Sports grounds offences possessing alcohol | 1 (0.02) | 7 (0.05) | 2 (0.02) |
| Threats and Extortion | 16 (0.36) | 24 (0.16) | 28 (0.30) |
| **Total recorded crimes** | **4,425** | **14,734** | **9,354** |

| **Table A5. Summary of weekly weekend night-time recorded crimes by cities** | | | | | | | | | |
| --- | --- | --- | --- | --- | --- | --- | --- | --- | --- |
| **City** | | **Year** | **Minimum** | **Maximum** | **Mean** | **SD** | **Median** | **IQR** | **Total** |
| **Interventions** | **Aberdeen** | 2015 | 10 | 42 | 21 | 7 | 20 | 8 | 748 |
|  |  | 2016 | 7 | 30 | 19 | 7 | 19 | 13 | 984 |
|  |  | 2017 | 7 | 54 | 19 | 8 | 17 | 9 | 972 |
|  |  | 2018 | 4 | 37 | 14 | 6 | 12 | 6 | 722 |
|  |  | 2019 | 5 | 33 | 16 | 6 | 16 | 7 | 845 |
|  |  | 2020 | 0 | 37 | 13 | 10 | 9 | 9 | 154 |
|  |  | **Total** | **0** | **54** | **17** | **7** | **16** | **9** | **4,425** |
|  | **Glasgow** | 2015 | 43 | 108 | 66 | 13 | 64 | 16 | 2,305 |
|  |  | 2016 | 28 | 90 | 60 | 14 | 61 | 14 | 3,118 |
|  |  | 2017 | 37 | 174 | 62 | 19 | 57 | 16 | 3,201 |
|  |  | 2018 | 33 | 86 | 53 | 10 | 52 | 13 | 2,760 |
|  |  | 2019 | 29 | 78 | 54 | 10 | 55 | 11 | 2,810 |
|  |  | 2020 | 16 | 70 | 45 | 14 | 45.5 | 17.5 | 540 |
|  |  | **Total** | **16** | **174** | **58** | **15** | **57** | **15** | **14,734** |
| **Control** | **Edinburgh** | 2015 | 23 | 83 | 44 | 12 | 42 | 13 | 1,536 |
|  |  | 2016 | 18 | 63 | 38 | 8 | 38 | 9 | 1,974 |
|  |  | 2017 | 24 | 107 | 38 | 12 | 36 | 12 | 1,992 |
|  |  | 2018 | 16 | 62 | 36 | 9 | 35 | 11 | 1,856 |
|  |  | 2019 | 18 | 53 | 32 | 7 | 32 | 9 | 1,660 |
|  |  | 2020 | 3 | 39 | 28 | 9 | 30 | 9 | 336 |
|  |  | **Total** | **3** | **107** | **37** | **10** | **36** | **12** | **9,354** |

**
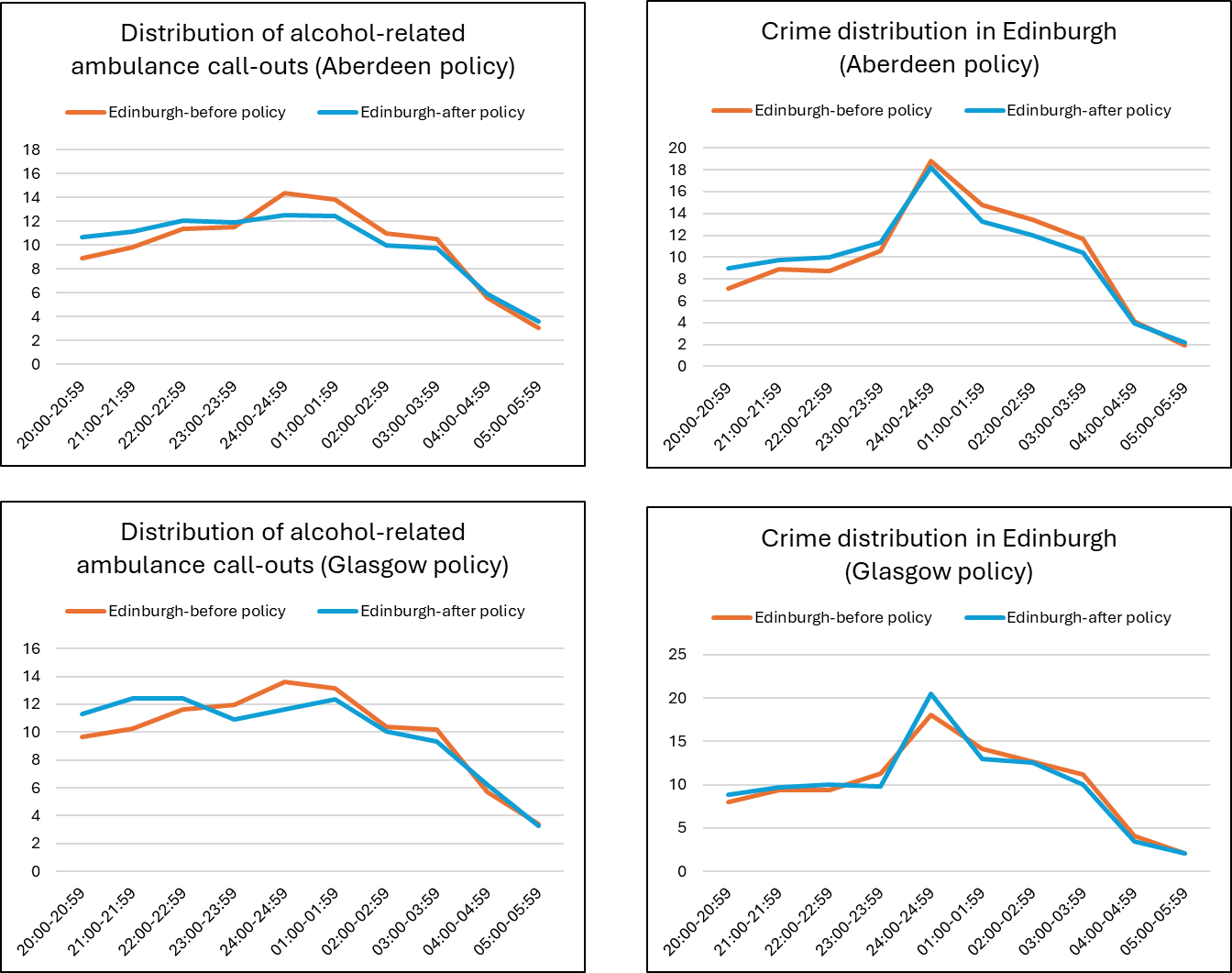
**

**Figure A2. Distribution of weekend night-time alcohol-related ambulance call-out and reported crimes in Edinburgh**

**
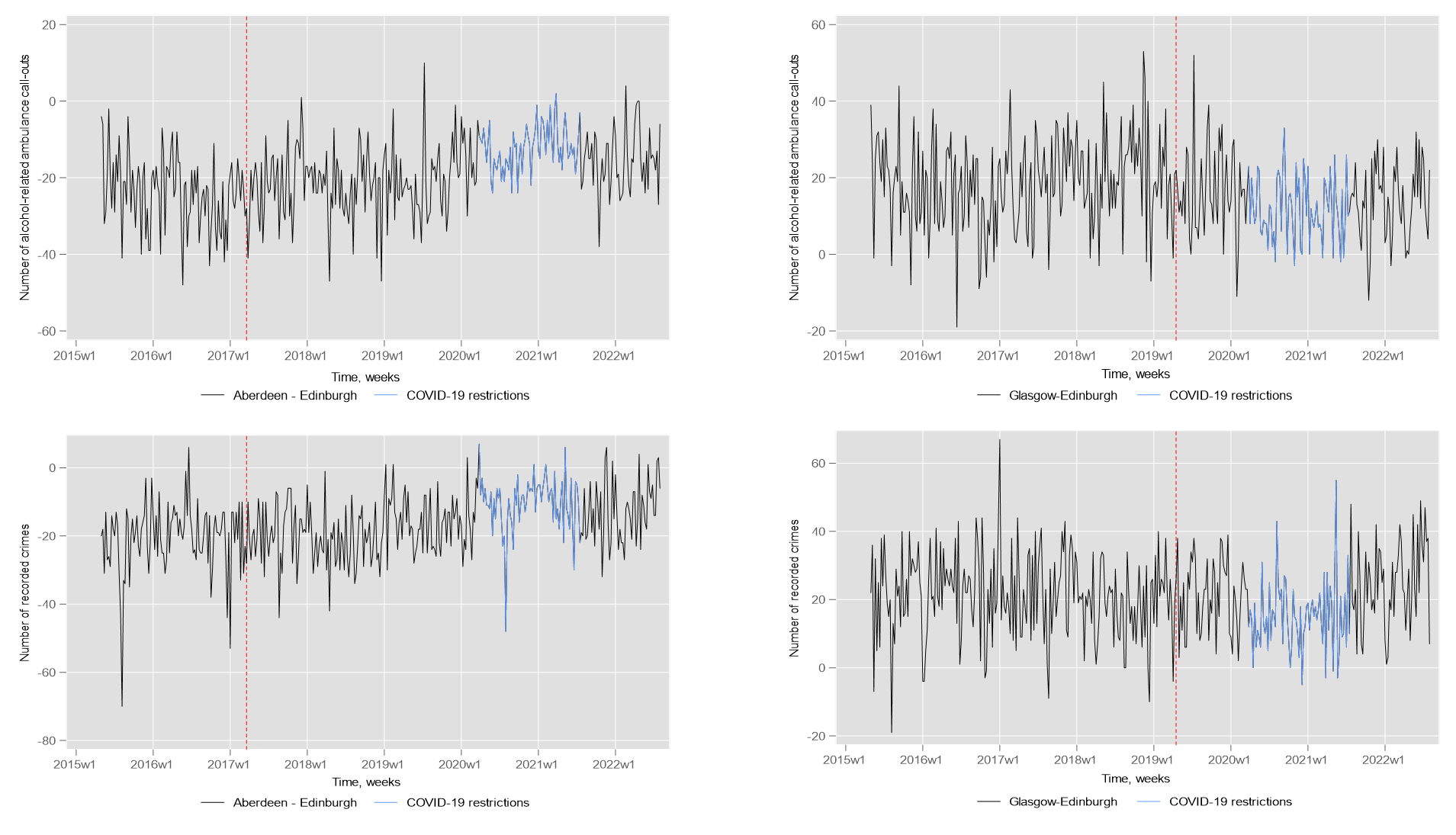
**

**Figure A3: Differences (intervention city minus (-) control city) of number of weekend night-time alcohol-related ambulance call-outs and reported crimes over-time between intervention and control cities**

## **Appendix 2.2. Description of confounding variables**

We included several confounding variables in our model, such as per capita gross disposable household income, weather conditions (mean temperature and rainfall), and the total number of on-premises alcohol outlets. These variables were incorporated as differences between the intervention and control areas (e.g., number of on-premises outlets in Aberdeen minus the number in Edinburgh). Additionally, we adjusted for dummy variables representing COVID-19 restrictions, public holidays, and outliers. These dummy variables were binary, taking the value “1” to indicate the presence of a restriction, holiday, or outlier, and “0” to indicate their absence. Public holiday data were extracted separately for each Scottish city. Outliers were defined as data points that fell more than 1.5 times the interquartile range above the upper quartile or below the lower quartile. To account for COVID-19 restrictions, we used city-specific lockdown policies based on the type of premises, as issued by the Scottish Government. While restrictions on bars, clubs, and nightclubs were implemented simultaneously across Scotland on 21st March 2020, those affecting nightclubs remained in place for a longer period compared to bars and pubs. We also included a time trend variable in the model to account for temporal effects, if it was statistically significant.

### ***Appendix 2.2.1. Data sources for confounding variables***

1. Per capita gross disposable household income ^1^
2. Weather conditions (mean temperature, rainfall) ^2^
3. Number of on-premises alcohol outlets ^3^
4. COVID-19 lockdown (imposing closure or restricted hours for alcohol-premises) ^4^
5. Public holidays ^5^

## **Appendix 2.3. Secondary and Sub-group analysis**

| **Table A6. Effect of policy changes on weekend restricted night-time (24:00 to 05:59) alcohol-related ambulance call-outs in Aberdeen and Glasgow** | | | | | | | | | |
| --- | --- | --- | --- | --- | --- | --- | --- | --- | --- |
| **Primary outcome, weekend restricted night-time (24:00 to 05:59) alcohol-related ambulance callouts** | **Main analysis** | | | **Sensitivity analysis** | | | | | |
|  | **Outcome: number of incidents** | | | **Outcome: number of incidents (extended time series, May 2015- July 2022)** | | | **Outcome: population adjusted incident rates** | | |
|  | **Coefficient** | **P-value** | **95% CI** | **Coefficient** | **P-value** | **95% CI** | **Coefficient** | **P-value** | **95% CI** |
| **Intervention area: Aberdeen** |  |  |  |  |  |  |  |  |  |
| **Staggered policy covariate** |  |  |  |  |  |  |  |  |  |
| Policy effect (all covariates) | **3.805** | 0.035 | 0.267, 7.343 | **3.674** | 0.000 | 1.796, 5.552 | 0.221 | 0.050 | 0.0002, 0.441 |
| Policy effect (significant covariates) | **3.970** | 0.021 | 0.609, 7.331 | **4.418** | 0.000 | 2.868, 5.969 | **0.143** | 0.003 | 0.048, 0.238 |
| **Dummy policy covariate, sensitivity analysis** |  |  |  |  |  |  |  |  |  |
| Policy effect (all covariates) | 2.950 | 0.070 | -0.235, 6.136 | **3.755** | 0.013 | 0.780, 6.731 | 0.097 | 0.204 | -0.052, 0.246 |
| Policy effect (significant covariates) | 2.140 | 0.316 | -2.041, 6.322 | **3.726** | 0.014 | 0.753, 6.700 | **0.090** | 0.011 | 0.021, 0.160 |
| **Dummy policy covariate with policy implemented with at least half strength, sensitivity analysis** |  |  |  |  |  |  |  |  |  |
| Policy effect (all covariates) | 2.286 | 0.103 | -0.459, 5.031 | 2.482 | 0.068 | -0.179, 5.143 | 0.043 | 0.450 | -0.069, 0.155 |
| Policy effect (significant covariates) | 2.352 | 0.068 | -0.172, 4.875 | **3.261** | 0.000 | 1.857, 4.666 | 0.075 | 0.084 | -0.010, 0.161 |
| **Intervention area: Glasgow** |  |  |  |  |  |  |  |  |  |
| **Dummy policy covariate** |  |  |  |  |  |  |  |  |  |
| Policy effect (all covariates) | 1.865 | 0.221 | -1.119, 4.849 | -0.996 | 0.453 | -3.600, 1.608 | 0.015 | 0.502 | -0.030, 0.061 |
| Policy effect (significant covariates) | 2.183 | 0.070 | -0.180, 4.546 | -1.269 | 0.139 | -2.947, 0.410 | 0.016 | 0.453 | -0.025, 0.056 |

| **Table A7. Effect of policy changes on weekend restricted night-time (20:00 to 23:59) alcohol-related ambulance call-outs in Aberdeen and Glasgow** | | | | | | | | | |
| --- | --- | --- | --- | --- | --- | --- | --- | --- | --- |
| **Primary outcome, weekend restricted night-time (20:00 to 23:59) alcohol-related ambulance callouts** | **Main analysis** | | | **Sensitivity analysis** | | | | | |
|  | **Outcome: number of incidents** | | | **Outcome: number of incidents (extended time series, May 2015- July 2022)** | | | **Outcome: population adjusted incident rates** | | |
|  | **Coefficient** | **P-value** | **95% CI** | **Coefficient** | **P-value** | **95% CI** | **Coefficient** | **P-value** | **95% CI** |
| **Intervention area: Aberdeen** |  |  |  |  |  |  |  |  |  |
| **Staggered policy covariate** |  |  |  |  |  |  |  |  |  |
| Policy effect (all covariates) | 0.497 | 0.631 | -1.528, 2.521 | **1.716** | 0.041 | 0.072, 3.360 | 0.104 | 0.099 | -0.020, 0.227 |
| Policy effect (significant covariates) | 0.440 | 0.669 | -1.573, 2.453 | **2.408** | 0.001 | 1.000, 3.816 | **0.079** | 0.005 | 0.024, 0.133 |
| **Dummy policy covariate, sensitivity analysis** |  |  |  |  |  |  |  |  |  |
| Policy effect (all covariates) | 0.304 | 0.823 | -2.365, 2.973 | -0.196 | 0.873 | -2.606, 2.214 | 0.025 | 0.524 | -0.052, 0.102 |
| Policy effect (significant covariates) | -0.014 | 0.983 | -1.365, 1.337 | -0.334 | 0.751 | -2.396, 1.728 | **0.046** | 0.014 | 0.009, 0.082 |
| **Dummy policy covariate with policy implemented with at least half strength, sensitivity analysis** |  |  |  |  |  |  |  |  |  |
| Policy effect (all covariates) | 0.093 | 0.935 | -2.135, 2.322 | **-2.118** | 0.038 | -4.115, -0.120 | -0.046 | 0.257 | -0.126, 0.034 |
| Policy effect (significant covariates) | -0.163 | 0.845 | -1.792, 1.466 | -2.040 | 0.113 | -4.564, 0.484 | -0.059 | 0.115 | -0.132, 0.014 |
| **Intervention area: Glasgow** |  |  |  |  |  |  |  |  |  |
| **Dummy policy covariate** |  |  |  |  |  |  |  |  |  |
| Policy effect (all covariates) | -2.281 | 0.182 | -5.630, 1.068 | -2.642 | 0.067 | -5.468, 0.183 | -0.032 | 0.175 | -0.078, 0.014 |
| Policy effect (significant covariates) | -2.183 | 0.066 | -4.513, 0.147 | **-3.218** | 0.000 | -4.701, -1.734 | -0.037 | 0.057 | -0.075, 0.001 |

| **Table A8. Effect of policy changes on weekend night-time all ambulance call-outs in Aberdeen and Glasgow** | | | | | | | | | |
| --- | --- | --- | --- | --- | --- | --- | --- | --- | --- |
| **Secondary outcome, weekend night-time all ambulance callouts** | **Main analysis** | | | **Sensitivity analysis** | | | | | |
|  | **Outcome: number of incidents** | | | **Outcome: number of incidents (extended time series, May 2015- July 2022)** | | | **Outcome: population adjusted incident rates** | | |
|  | **Coefficient** | **P-value** | **95% CI** | **Coefficient** | **P-value** | **95% CI** | **Coefficient** | **P-value** | **95% CI** |
| **Intervention area: Aberdeen** |  |  |  |  |  |  |  |  |  |
| **Staggered policy covariate** |  |  |  |  |  |  |  |  |  |
| Policy effect (all covariates) | 6.373 | 0.139 | -2.070, 14.815 | **11.761** | 0.005 | 3.550, 19.972 | 0.369 | 0.057 | -0.012, 0.750 |
| Policy effect (significant covariates) | 6.055 | 0.161 | -2.406, 14.517 | **12.895** | 0.000 | 6.003, 19.787 | **0.312** | 0.005 | 0.093, 0.532 |
| **Dummy policy covariate, sensitivity analysis** |  |  |  |  |  |  |  |  |  |
| Policy effect (all covariates) | 4.204 | 0.134 | -1.297, 9.704 | 5.788 | 0.197 | -3.011, 14.587 | 0.137 | 0.301 | -0.123, 0.396 |
| Policy effect (significant covariates) | 3.997 | 0.157 | -1.543, 9.537 | 7.337 | 0.102 | -1.469, 16.143 | **0.173** | 0.034 | 0.013, 0.333 |
| **Dummy policy covariate with policy implemented with at least half strength, sensitivity analysis** |  |  |  |  |  |  |  |  |  |
| Policy effect (all covariates) | 5.294 | 0.083 | -0.683, 11.272 | **8.816** | 0.010 | 2.119, 15.512 | 0.128 | 0.154 | -0.048, 0.304 |
| Policy effect (significant covariates) | 4.593 | 0.122 | -1.234, 10.419 | **9.704** | 0.001 | 4.050, 15.358 | **0.179** | 0.026 | 0.021, 0.338 |
| **Intervention area: Glasgow** |  |  |  |  |  |  |  |  |  |
| **Dummy policy covariate** |  |  |  |  |  |  |  |  |  |
| Policy effect (all covariates) | -3.437 | 0.468 | -12.711, 5.836 | -5.843 | 0.200 | -14.787, 3.102 | -0.018 | 0.849 | -0.204, 0.168 |
| Policy effect (significant covariates) | -1.266 | 0.763 | -9.509, 6.976 | -5.990 | 0.084 | -12.779, 0.798 | 0.031 | 0.742 | -0.152, 0.214 |

| **Table A9. Effect of policy changes on weekend night-time alcohol-related ambulance call-outs in Aberdeen and Glasgow among female sub-group** | | | | | | | | | |
| --- | --- | --- | --- | --- | --- | --- | --- | --- | --- |
| **Primary outcome, weekend night-time alcohol-related ambulance callouts** | **Main analysis** | | | **Sensitivity analysis** | | | | | |
|  | **Outcome: number of incidents** | | | **Outcome: number of incidents (extended time series, May 2015- July 2022)** | | | **Outcome: population adjusted incident rates** | | |
|  | **Coefficient** | **P-value** | **95% CI** | **Coefficient** | **P-value** | **95% CI** | **Coefficient** | **P-value** | **95% CI** |
| **Intervention area: Aberdeen** |  |  |  |  |  |  |  |  |  |
| **Staggered policy covariate** |  |  |  |  |  |  |  |  |  |
| Policy effect (all covariates) | 8.601 | 0.012 | 1.856, 15.346 | **2.481** | 0.006 | 0.724, 4.237 | **0.083** | 0.041 | 0.003, 0.162 |
| Policy effect (significant covariates) | 2.009 | 0.044 | 0.058, 3.959 | **3.661** | 0.000 | 2.285, 5.037 | **0.086** | 0.001 | 0.037, 0.135 |
| **Dummy policy covariate, sensitivity analysis** |  |  |  |  |  |  |  |  |  |
| Policy effect (all covariates) | 0.928 | 0.168 | -0.392, 2.248 | 0.988 | 0.162 | -0.396, 2.372 | 0.038 | 0.231 | -0.024, 0.099 |
| Policy effect (significant covariates) | 0.974 | 0.152 | -0.358, 2.307 | 0.980 | 0.168 | -0.413, 2.373 | **0.051** | 0.002 | 0.019, 0.083 |
| **Dummy policy covariate with policy implemented with at least half strength, sensitivity analysis** |  |  |  |  |  |  |  |  |  |
| Policy effect (all covariates) | 1.180 | 0.158 | -0.457, 2.818 | 1.516 | 0.059 | -0.057, 3.090 | 0.023 | 0.270 | -0.018, 0.064 |
| Policy effect (significant covariates) | 1.149 | 0.148 | -0.407, 2.705 | **1.932** | 0.013 | 0.414, 3.450 | 0.023 | 0.260 | -0.017, 0.064 |
| **Intervention area: Glasgow** |  |  |  |  |  |  |  |  |  |
| **Dummy policy covariate** |  |  |  |  |  |  |  |  |  |
| Policy effect (all covariates) | -2.060 | 0.107 | -4.565, 0.444 | -1.597 | 0.097 | -3.485, 0.291 | -0.022 | 0.237 | -0.059, 0.015 |
| Policy effect (significant covariates) | **-2.491** | 0.009 | -4.371, -0.611 | **-2.104** | 0.000 | -3.070, -1.139 | **-0.038** | 0.009 | -0.067, -0.010 |

| **Table A10. Effect of policy changes on weekend night-time alcohol-related ambulance call-outs in Aberdeen and Glasgow among male sub-group** | | | | | | | | | |
| --- | --- | --- | --- | --- | --- | --- | --- | --- | --- |
| **Primary outcome, weekend night-time alcohol-related ambulance callouts** | **Main analysis** | | | **Sensitivity analysis** | | | | | |
|  | **Outcome: number of incidents** | | | **Outcome: number of incidents (extended time series, May 2015- July 2022)** | | | **Outcome: population adjusted incident rates** | | |
|  | **Coefficient** | **P-value** | **95% CI** | **Coefficient** | **P-value** | **95% CI** | **Coefficient** | **P-value** | **95% CI** |
| **Intervention area: Aberdeen** |  |  |  |  |  |  |  |  |  |
| **Staggered policy covariate** |  |  |  |  |  |  |  |  |  |
| Policy effect (all covariates) | 4.045 | 0.001 | 1.552, 6.537 | **3.401** | 0.001 | 1.357, 5.444 | 0.071 | 0.183 | -0.033, 0.175 |
| Policy effect (significant covariates) | 3.995 | 0.002 | 1.487, 6.502 | **3.726** | 0.000 | 1.946, 5.507 | **0.115** | 0.002 | 0.041, 0.189 |
| **Dummy policy covariate, sensitivity analysis** |  |  |  |  |  |  |  |  |  |
| Policy effect (all covariates) | 2.336 | 0.004 | 0.734, 3.938 | **1.943** | 0.019 | 0.321, 3.566 | 0.076 | 0.07 | -0.006, 0.159 |
| Policy effect (significant covariates) | 2.261 | 0.017 | 0.404, 4.119 | **1.977** | 0.022 | 0.280, 3.673 | **0.090** | 0.001 | 0.039, 0.142 |
| **Dummy policy covariate with policy implemented with at least half strength, sensitivity analysis** |  |  |  |  |  |  |  |  |  |
| Policy effect (all covariates) | 2.114 | 0.040 | 0.098, 4.131 | **1.894** | 0.025 | 0.243, 3.545 | 0.014 | 0.653 | -0.047, 0.074 |
| Policy effect (significant covariates) | 1.818 | 0.077 | -0.200, 3.835 | **2.315** | 0.002 | 0.815, 3.816 | 0.007 | 0.816 | -0.055, 0.069 |
| **Intervention area: Glasgow** |  |  |  |  |  |  |  |  |  |
| **Dummy policy covariate** |  |  |  |  |  |  |  |  |  |
| Policy effect (all covariates) | -0.395 | 0.780 | -3.162, 2.372 | 2.392 | 0.300 | -2.131, 6.916 | -0.007 | 0.747 | -0.051, 0.037 |
| Policy effect (significant covariates) | -2.150 | 0.053 | -4.330, 0.029 | 1.463 | 0.500 | -2.783, 5.709 | 0.017 | 0.368 | -0.020, 0.053 |

| **Table A11. Effect of policy changes on weekend night-time alcohol-related ambulance call-outs in Aberdeen and Glasgow among patients age less than 45 years** | | | | | | | | | | | |
| --- | --- | --- | --- | --- | --- | --- | --- | --- | --- | --- | --- |
| **Primary outcome, weekend night-time alcohol-related ambulance callouts** | **Main analysis** | | | **Sensitivity analysis** | | | | | | |  |
|  | **Outcome: number of incidents** | | | **Outcome: number of incidents (extended time series, May 2015- July 2022)** | | | **Outcome: population adjusted incident rates** | | |  |  |
|  | **Coefficient** | **P-value** | **95% CI** | **Coefficient** | **P-value** | **95% CI** | **Coefficient** | **P-value** | **95% CI** |  |  |
| **Intervention area: Aberdeen** |  |  |  |  |  |  |  |  |  |  |  |
| **Staggered policy covariate** |  |  |  |  |  |  |  |  |  |  |  |
| Policy effect (all covariates) | **15.626** | 0.000 | 10.292, 20.959 | **6.180** | 0.002 | 2.251, 10.109 | **0.270** | 0.000 | 0.125, 0.416 |  |  |
| Policy effect (significant covariates) | **14.904** | 0.000 | 9.983, 19.825 | **7.050** | 0.000 | 4.077, 10.023 | **0.153** | 0.000 | 0.090, 0.216 |  |  |
| **Dummy policy covariate, sensitivity analysis** |  |  |  |  |  |  |  |  |  |  |  |
| Policy effect (all covariates) | 1.897 | 0.406 | -2.577, 6.371 | 1.527 | 0.648 | -5.026, 8.080 | 0.054 | 0.261 | -0.040, 0.147 |  |  |
| Policy effect (significant covariates) | 1.860 | 0.390 | -2.384, 6.104 | 1.355 | 0.705 | -5.670, 8.380 | 0.057 | 0.198 | -0.030, 0.145 |  |  |
| **Dummy policy covariate with policy implemented with at least half strength, sensitivity analysis** |  |  |  |  |  |  |  |  |  |  |  |
| Policy effect (all covariates) | 2.821 | 0.245 | -1.936, 7.577 | 1.500 | 0.873 | -16.828, 19.827 | 0.022 | 0.795 | -0.142, 0.185 |  |  |
| Policy effect (significant covariates) | 2.595 | 0.179 | -1.188, 6.378 | 3.732 | 0.562 | -8.876, 16.340 | 0.040 | 0.619 | -0.119, 0.200 |  |  |
| **Intervention area: Glasgow** |  |  |  |  |  |  |  |  |  |  |  |
| **Dummy policy covariate** |  |  |  |  |  |  |  |  |  |  |  |
| Policy effect (all covariates) | 0.908 | 0.577 | -2.282, 4.099 | -0.889 | 0.516 | -3.575, 1.797 | 0.003 | 0.906 | -0.051, 0.057 |  |  |
| Policy effect (significant covariates) | 2.250 | 0.110 | -0.510, 5.010 | **-2.543** | 0.014 | -4.579, -0.506 | 0.001 | 0.968 | -0.048, 0.050 |  |  |

| **Table A12. Effect of policy changes on weekend night-time alcohol-related ambulance call-outs in Aberdeen and Glasgow among patients age more than 45 years** | | | | | | | | | | |
| --- | --- | --- | --- | --- | --- | --- | --- | --- | --- | --- |
| **Primary outcome, weekend night-time alcohol-related ambulance callouts** | **Main analysis** | | | **Sensitivity analysis** | | | | | |  |
|  | **Outcome: number of incidents** | | | **Outcome: number of incidents (extended time series, May 2015- July 2022)** | | | **Outcome: population adjusted incident rates** | | |  |
|  | **Coefficient** | **P-value** | **95% CI** | **Coefficient** | **P-value** | **95% CI** | **Coefficient** | **P-value** | **95% CI** |  |
| **Intervention area: Aberdeen** |  |  |  |  |  |  |  |  |  |  |
| **Staggered policy covariate** |  |  |  |  |  |  |  |  |  |  |
| Policy effect (all covariates) | 0.315 | 0.877 | -3.664, 4.295 | 0.891 | 0.689 | -3.478, 5.261 | -0.006 | 0.933 | -0.152, 0.140 |  |
| Policy effect (significant covariates) | 0.357 | 0.681 | -1.346, 2.060 | **0.806** | 0.009 | 0.203, 1.408 | 0.062 | 0.059 | -0.002, 0.126 |  |
| **Dummy policy covariate, sensitivity analysis** |  |  |  |  |  |  |  |  |  |  |
| Policy effect (all covariates) | 0.886 | 0.355 | -0.992, 2.764 | 0.925 | 0.288 | -0.783, 2.633 | 0.018 | 0.599 | -0.050, 0.086 |  |
| Policy effect (significant covariates) | 0.499 | 0.365 | -0.580, 1.578 | **0.679** | 0.008 | 0.181, 1.178 | **0.045** | 0.029 | 0.005, 0.086 |  |
| **Dummy policy covariate with policy implemented with at least half strength, sensitivity analysis** |  |  |  |  |  |  |  |  |  |  |
| Policy effect (all covariates) | -0.341 | 0.697 | -2.057, 1.376 | 0.163 | 0.857 | -1.616, 1.943 | 0.009 | 0.776 | -0.053, 0.072 |  |
| Policy effect (significant covariates) | 0.043 | 0.948 | -1.243, 1.328 | 0.571 | 0.053 | -0.008, 1.150 | 0.036 | 0.124 | -0.010, 0.081 |  |
| **Intervention area: Glasgow** |  |  |  |  |  |  |  |  |  |  |
| **Dummy policy covariate** |  |  |  |  |  |  |  |  |  |  |
| Policy effect (all covariates) | **-4.629** | 0.000 | -6.691, -2.567 | -1.139 | 0.458 | -4.144, 1.867 | -0.029 | 0.067 | -0.061, 0.002 |  |
| Policy effect (significant covariates) | **-3.452** | 0.000 | -4.713, -2.190 | -1.566 | 0.293 | -4.484, 1.352 | **-0.037** | 0.009 | -0.065, -0.009 |  |

| **Table A13. Effect of policy changes on weekend restricted night-time (24:00 to 05:59) recorded crimes in Aberdeen and Glasgow** | | | | | | | | | |
| --- | --- | --- | --- | --- | --- | --- | --- | --- | --- |
| **Secondary outcome, weekend restricted night-time (24:00 to 05:59) recorded crimes** | **Main analysis** | | | **Sensitivity analysis** | | | | | |
|  | **Outcome: number of incidents** | | | **Outcome: number of incidents (extended time series, May 2015- July 2022)** | | | **Outcome: population adjusted incident rates** | | |
|  | **Coefficient** | **P-value** | **95% CI** | **Coefficient** | **P-value** | **95% CI** | **Coefficient** | **P-value** | **95% CI** |
| **Intervention area: Aberdeen** | | | | | | | | | |
| **Staggered policy covariate** |  |  |  |  |  |  |  |  |  |
| Policy effect (all covariates) | 3.752 | 0.179 | -1.721, 9.226 | 4.534 | 0.071 | -0.385, 9.454 | **0.159** | 0.008 | 0.042, 0.277 |
| Policy effect (significant covariates) | **3.483** | 0.003 | 1.153, 5.814 | **2.853** | 0.002 | 1.073, 4.634 | **0.191** | 0.000 | 0.101, 0.282 |
| **Dummy policy covariate, sensitivity analysis** |  |  |  |  |  |  |  |  |  |
| Policy effect (all covariates) | -0.062 | 0.970 | -3.239, 3.116 | -0.060 | 0.967 | -2.917, 2.797 | -0.039 | 0.393 | -0.128, 0.050 |
| Policy effect (significant covariates) | **1.805** | 0.033 | 0.150, 3.460 | **1.572** | 0.028 | 0.174, 2.970 | -0.038 | 0.384 | -0.123, 0.047 |
| **Dummy policy covariate with policy implemented with at least half strength, sensitivity analysis** |  |  |  |  |  |  |  |  |  |
| Policy effect (all covariates) | **2.291** | 0.042 | 0.079, 4.503 | **2.757** | 0.007 | 0.759, 4.755 | **0.075** | 0.000 | 0.035, 0.114 |
| Policy effect (significant covariates) | **2.821** | 0.001 | 1.171, 4.472 | **2.642** | 0.000 | 1.271, 4.013 | **0.069** | 0.000 | 0.031, 0.107 |
| **Intervention area: Glasgow** | | | | | | | | | |
| **Dummy policy covariate** |  |  |  |  |  |  |  |  |  |
| Policy effect (all covariates) | -1.869 | 0.280 | -5.256, 1.518 | **-6.301** | 0.003 | -10.423, -2.178 | **-0.099** | 0.029 | -0.187, -0.010 |
| Policy effect (significant covariates) | -0.625 | 0.654 | -3.356, 2.106 | **-5.863** | 0.003 | -9.687, -2.040 | **-0.084** | 0.048 | -0.167, -0.001 |

## **Appendix 2.4: Falsification test**

| **Table A14. Falsification test for weekend night-time alcohol-related ambulance call-outs and all ambulance call-outs in Aberdeen and Glasgow** | | | | | | | | | | | | | |
| --- | --- | --- | --- | --- | --- | --- | --- | --- | --- | --- | --- | --- | --- |
|  | **Primary outcome: weekend night-time alcohol-related ambulance call-outs** | | | **Primary outcome: weekend restricted night-time (24:00 to 05:59) alcohol-related ambulance call-outs** | | | **Primary outcome: weekend restricted night-time (20:00 to 23:59) alcohol-related ambulance call-outs** | | | **Secondary outcome: weekend night-time all ambulance call-outs** | | |  |
|  | **Falsification test, 1 year before policy implementation, (outcome: number of incidents)** | | | **Falsification test, 1 year before policy implementation, (outcome: number of incidents)** | | | **Falsification test, 1 year before policy implementation, (outcome: number of incidents)** | | | **Falsification test, 1 year before policy implementation, (outcome: number of incidents)** | | |  |
|  | **Coefficient** | **P-value** | **95% CI** | **Coefficient** | **P-value** | **95% CI** | **Coefficient** | **P-value** | **95% CI** | **Coefficient** | **P-value** | **95% CI** |  |
| **Intervention area: Aberdeen** | | | | | | | | | |  |  |  |  |
| **Staggered policy covariate** |  |  |  |  |  |  |  |  |  |  |  |  |  |
| Policy effect (all covariates) | -16.911 | 0.098 | -36.918, 3.097 | 2.308 | 0.321 | -2.254, 6.871 | **-12.984** | 0.011 | -23.016, -2.951 | **-37.714** | 0.024 | -70.452, -4.977 |  |
| Policy effect (significant covariates) | -13.762 | 0.170 | -33.396, 5.873 | 2.693 | 0.179 | -1.236, 6.622 | **-11.051** | 0.028 | -20.925, -1.177 | -31.296 | 0.057 | -63.469, 0.878 |  |
| **Dummy policy covariate, sensitivity analysis** |  |  |  |  |  |  |  |  |  |  |  |  |  |
| Policy effect (all covariates) | -4.806 | 0.105 | -10.609, 0.998 | -0.072 | 0.975 | -4.548, 4.404 | -1.713 | 0.226 | -4.486, 1.059 | -7.185 | 0.170 | 17.444, 3.075 |  |
| Policy effect (significant covariates) | -3.718 | 0.161 | -8.921, 1.485 | 1.011 | 0.616 | -2.935, 4.957 | -0.747 | 0.377 | -2.404, 0.911 | -7.278 | 0.111 | -16.236, 1.681 |  |
| **Dummy policy covariate with policy implemented with at least half strength, sensitivity analysis** |  |  |  |  |  |  |  |  |  |  |  |  |  |
| Policy effect (all covariates) | 2.035 | 0.193 | -1.032, 5.102 | 1.564 | 0.331 | -1.592, 4.721 | 0.109 | 0.966 | -4.926, 5.144 | -6.141 | 0.354 | -19.116, 6.833 |  |
| Policy effect (significant covariates) | 2.060 | 0.153 | -0.765, 4.885 | 1.220 | 0.404 | -1.646, 4.087 | 0.096 | 0.892 | -1.284, 1.476 | 1.877 | 0.478 | -3.304, 7.058 |  |
| **Intervention area: Glasgow** | | | | | | | | | |  |  |  |  |
| **Dummy policy covariate** |  |  |  |  |  |  |  |  |  |  |  |  |  |
| Policy effect (all covariates) | 1.639 | 0.586 | -4.264, 7.542 | -0.351 | 0.859 | -4.231, 3.529 | 2.769 | 0.133 | -0.841, 6.379 | -1.567 | 0.783 | -12.719, 9.584 |  |
| Policy effect (significant covariates) | 1.089 | 0.480 | -1.931, 4.109 | 0.764 | 0.462 | -1.272, 2.800 | 0.628 | 0.537 | -1.365, 2.622 | 5.560 | 0.054 | -0.087, 11.207 |  |

| **Table A15. Falsification test for sub-group analysis for the weekend night-time alcohol-related ambulance call-outs in Aberdeen and Glasgow** | | | | | | | | | | | | |
| --- | --- | --- | --- | --- | --- | --- | --- | --- | --- | --- | --- | --- |
|  | **Sub-group: Female** | | | **Sub-group: Male** | | | **Sub-group: age less than 45 years** | | | **Sub-group: age more than 45 years** | | |
|  | **Falsification test, 1 year before policy implementation, (outcome: number of incidents)** | | | **Falsification test, 1 year before policy implementation, (outcome: number of incidents)** | | | **Falsification test, 1 year before policy implementation, (outcome: number of incidents)** | | | **Falsification test, 1 year before policy implementation, (outcome: number of incidents)** | | |
|  | **Coefficient** | **P-value** | **95% CI** | **Coefficient** | **P-value** | **95% CI** | **Coefficient** | **P-value** | **95% CI** | **Coefficient** | **P-value** | **95% CI** |
| **Intervention area: Aberdeen** | | | | | | | | | | | | |
| **Staggered policy covariate** |  |  |  |  |  |  |  |  |  |  |  |  |
| Policy effect (all covariates) | -7.281 | 0.083 | -15.524, 0.961 | -7.702 | 0.191 | -19.251, 3.847 | -14.762 | 0.087 | -31.683, 2.159 | 0.893 | 0.752 | -4.655, 6.441 |
| Policy effect (significant covariates) | -6.855 | 0.097 | -14.948, 1.239 | **2.961** | 0.013 | 0.617, 5.305 | -12.503 | 0.097 | -27.263, 2.256 | 0.431 | 0.575 | -1.074, 1.935 |
| **Dummy policy covariate, sensitivity analysis** |  |  |  |  |  |  |  |  |  |  |  |  |
| Policy effect (all covariates) | -2.348 | 0.058 | -4.781, 0.084 | -2.577 | 0.144 | -6.036, 0.882 | -4.095 | 0.075 | -8.609, 0.420 | -0.812 | 0.427 | -2.814, 1.191 |
| Policy effect (significant covariates) | -1.435 | 0.200 | -3.633, 0.762 | -2.405 | 0.110 | -5.358, 0.547 | -3.295 | 0.087 | -7.064, 0.475 | 0.279 | 0.666 | -0.989, 1.548 |
| **Dummy policy covariate with policy implemented with at least half strength, sensitivity analysis** |  |  |  |  |  |  |  |  |  |  |  |  |
| Policy effect (all covariates) | **-4.067** | 0.038 | -7.903, -0.232 | 0.918 | 0.723 | -4.165, 6.002 | 2.450 | 0.152 | -0.904, 5.804 | 0.483 | 0.755 | -2.545, 3.511 |
| Policy effect (significant covariates) | **-4.178** | 0.031 | -7.977, -0.379 | 1.671 | 0.291 | -1.433, 4.775 | 2.523 | 0.104 | -0.516, 5.563 | 0.045 | 0.936 | -1.050, 1.140 |
| **Intervention area: Glasgow** | | | | | | | | | | | | |
| **Dummy policy covariate** |  |  |  |  |  |  |  |  |  |  |  |  |
| Policy effect (all covariates) | -1.767 | 0.265 | -4.875, 1.341 | **3.846** | 0.038 | 0.219, 7.472 | 3.496 | 0.079 | -0.399, 7.391 | -0.609 | 0.648 | -3.219, 2.002 |
| Policy effect (significant covariates) | **-1.575** | 0.028 | -2.983, -0.167 | **4.877** | 0.004 | 1.564, 8.190 | 2.473 | 0.002 | 0.908, 4.037 | -0.703 | 0.288 | -2.000, 0.594 |

| **Table A16. Falsification test for weekend night-time recorded crimes in Aberdeen and Glasgow** | | | | | | |
| --- | --- | --- | --- | --- | --- | --- |
|  | **Secondary outcome, weekend night-time recorded crimes** | | | **Secondary outcome, weekend restricted night-time (24:00 to 05:59) recorded crimes** | | |
|  | **Falsification test, 1 year before policy implementation, (outcome: number of incidents)** | | | **Falsification test, 1 year before policy implementation, (outcome: number of incidents)** | | |
|  | **Coefficient** | **P-value** | **95% CI** | **Coefficient** | **P-value** | **95% CI** |
| **Intervention area: Aberdeen** | | | | | | |
| **Staggered policy covariate** |  |  |  |  |  |  |
| Policy effect (all covariates) | -1.772 | 0.776 | -14.006, 10.461 | -2.278 | 0.653 | -12.201, 7.644 |
| Policy effect (significant covariates) | 2.407 | 0.123 | -0.653, 5.467 | **2.661** | 0.016 | 0.497, 4.825 |
| **Dummy policy covariate, sensitivity analysis** |  |  |  |  |  |  |
| Policy effect (all covariates) | -1.382 | 0.562 | -6.056, 3.292 | -1.579 | 0.370 | -5.029, 1.872 |
| Policy effect (significant covariates) | 1.326 | 0.426 | -1.937, 4.590 | -1.432 | 0.422 | -4.925, 2.061 |
| **Dummy policy covariate with policy implemented with at least half strength, sensitivity analysis** |  |  |  |  |  |  |
| Policy effect (all covariates) | -2.017 | 0.568 | -8.933, 4.899 | -1.740 | 0.537 | -7.262, 3.781 |
| Policy effect (significant covariates) | 0.574 | 0.740 | -2.812, 3.960 | **-3.440** | 0.018 | -6.295, -0.586 |
| **Intervention area: Glasgow** | | | | | | |
| **Dummy policy covariate** |  |  |  |  |  |  |
| Policy effect (all covariates) | **-3.790** | 0.03 | -7.219, -0.361 | **-4.844** | 0.009 | -8.492, -1.196 |
| Policy effect (significant covariates) | **-2.712** | 0.000 | -4.042, -1.382 | **-3.766** | 0.000 | -5.551, -1.982 |

# **Appendix 3. Synthetic control**

## **Appendix 3.1. Alcohol-related ambulance call-outs (Aberdeen)**

### ***Appendix 3.1.1. Model specification and validation test***

The analysis of the synthetic control was conducted in two main steps. First, we assessed nine different model specifications derived from the existing literature on synthetic control methods, following the recommended validation procedure for synthetic control analysis (Table SC 1) ^6,7^. The validation test involved calculating the mean squared prediction error (MSPE) by splitting the pre-intervention periods into training and validation sets. Since there are no recommended ratios for splitting the pre-intervention period, we tested three different split ratios—50:50, 70:30, and 80:20—and compared MSPEs across the different specifications. For the alcohol-related ambulance call-outs in Aberdeen, we identified three specifications (1, 2, and 3 (see below Table SC 1)) that produced the lowest MSPEs. However, literature recommends against using all pre-intervention outcome values as covariates, as this can reduce the predictive power of other variables that may significantly influence the construction of the counterfactual ^7,8^. Therefore, we selected specification 3 for the remainder of the synthetic control analysis. This specification was chosen because it struck a balance between capturing the pre-intervention trends and allowing other important covariates to contribute to the counterfactual.

### ***Appendix 3.1.2. Main analysis and sensitivity tests***

For the analysis of alcohol-related ambulance call-outs, we observed that the predictor means for Aberdeen were closely aligned with those of the synthetic Aberdeen, as opposed to the average of all donor pool/control cities (Table SC 2). The number of on-premises alcohol outlets emerged as the most influential covariate in generating the synthetic control weights, alongside the pre-intervention outcome mean. Among the donor pools, Edinburgh was the most significant contributor to the creation of synthetic Aberdeen, followed by North Lanarkshire and East Renfrewshire (Figure SC 01).

From the gap plot between Aberdeen and synthetic Aberdeen, a positive average treatment effect (ATT=0·095) appeared evident (Figure SC 02). However, a placebo test using permutation methods indicated that other council areas might also exhibit a positive ATT although no policy change occurred there (Figure SC 03a). As a sensitivity analysis, we also presented two graphs for the placebo test after excluding council areas with MSPE two times higher and one time higher than Aberdeen's (Figures SC 03b and SC 03c). This exclusion aimed to improve the robustness of the comparison.

For Aberdeen, we calculated the ATT by accounting for the staggered implementation of the policy, adjusting the ATT accordingly using staggered policy weights. Additionally, we visualised the distribution of effect sizes (i.e., ATT) based on the placebo test, where the second figure only considered positive effect sizes, further narrowing the focus to more relevant comparisons (Figures SC 04a and SC 04b). This distribution provides a clear depiction of how extreme the ATT for Aberdeen is compared to the control/donor cities. From the figures, we observed that while some control units exhibited positive ATTs, the magnitude of Aberdeen’s ATT was comparatively larger. This suggests that the treatment effect in Aberdeen is more pronounced than in most control cities, strengthening the uniqueness of the observed impact in Aberdeen relative to the donor pool.

Finally, we compared post/pre-intervention MSPE ratios for Aberdeen and the control cities and calculated a frequentist p-value. This was done by dividing the number of cities with post/pre MSPE ratios higher than Aberdeen's by the total number of cities. This resulted in a p-value of 0·222 (6/27) (Figure SC 05a). The second figure illustrating post/pre MSPE ratios was also presented, this time only considering cities with positive effect sizes, enhancing the interpretability of the results with a focus on potential treatment effects (Figure SC 05b). The estimated p-value in this analysis was 0·28, calculated as 5/18 (Figure SC 05b). It is important to note that the minimum possible p-value in synthetic control studies depends on the number of control units included in the donor pool. For instance, with 27 control units, the minimum attainable p-value would be 1/27 (approximately 0·04). In contrast, if 18 control units are used, the minimum p-value would be 1/18, (approximately 0·06). Thus, the number of control units directly influences the lowest possible p-value that can be observed. In addition, we conducted ARIMA based on difference-in-difference analyses with the synthetic control where we modelled the differences of alcohol-related ambulance call-out rates between Aberdeen and Synthetic Aberdeen ^9^. We estimated effect size of 0·205 with a p-value <0·001 (Table A17).

| **Table SC 1. Validation test for synthetic control for alcohol-related ambulance call-outs in Aberdeen** | | | |
| --- | --- | --- | --- |
| **Specifications** | **Pre-intervention periods split between training and validation** | | |
|  | **Training/validation, 50/50** | **Training/validation, 70/30** | **Training/validation, 80/20** |
|  | **MSPE* (Validation)** | **MSPE* (Validation)** | **MSPE* (Validation)** |
| 1. (all pre-intervention outcome values and covariates) | 0·0336 | 0·03522 | 0·0480 |
| 2. (all pre-intervention outcome values only) | 0·0335 | 0·03524 | 0·0479 |
| 3. (pre-intervention outcome mean and covariates) | 0·0412 | 0·0384 | 0·0435 |
| 4. (last pre-intervention outcome value and covariates) | 0·0807 | 0·1267 | 0·1002 |
| 5. (pre-intervention outcome mean and last pre-intervention outcome value, and covariates) | 0·0404 | 0·0389 | 0·0473 |
| 6. (first three-fourths of the pre-intervention outcome values and covariates) | 0·0370 | 0·0411 | 0·0503 |
| 7. (last three-fourths of the pre-intervention outcome values and covariates) | 0·0356 | 0·0386 | 0·0528 |
| 8. (odd pre-intervention outcome values and covariates) | 0·0359 | 0·0388 | 0·0516 |
| 9. (even pre-intervention outcome values and covariates) | 0·0367 | 0·0402 | 0·0458 |

***Mean Square Prediction Error**

**Aberdeen- Synthetic Aberdeen, Specification 3:**

| **Table SC 2. Covariate balance in the pre-intervention periods** | | | | |  | |
| --- | --- | --- | --- | --- | --- | --- |
| **Covariates** | **Covariate weights** | **Alcohol-related ambulance call-outs, predictor means** | | | |  |
|  |  | **Aberdeen** | **Synthetic Aberdeen** | **Average of other control cities** | |  |
| Number of on-premises alcohol outlets (population size adjusted) | 0·696 | 19·813 | 20·324 | 23·554 | |  |
| Per capita gross disposable household income | 0·045 | 20,125 | 19,390 | 17,763 | |  |
| Pre-intervention outcome mean | 0·259 | 0·724 | 0·712 | 0·538 | |  |

**Figure SC 01: Optimal unit weights**

**Figure SC 02: Gaps of Aberdeen and synthetic Aberdeen**

**Figure SC 03a: Placebo test**

**Figure SC 03b: Placebo test excluding control cities MSPE>2 times Aberdeen**

**Figure SC 03c: Placebo test excluding control cities if MSPE> Aberdeen**

**Figure SC 04a: Effect size for placebo test**

**Figure** **SC 04b: Effect size placebo test considering only positive effect size**

**Figure SC 05a: Post/Pre MSPE ratio across cities**

**Figure SC 05b:** **Post/Pre MSPE ratio across cities considering only positive effect size**

## **Appendix 3.2. Alcohol-related ambulance call-outs (Glasgow)**

### ***Appendix 3.2.1. Model specification and validation test***

For the analysis of alcohol-related ambulance call-outs in Glasgow, we identified two specifications (3 and 7) that yielded the lowest MSPEs (Table SC 3). Specification 3 produced the smallest MSPE on two occasions and had the lowest overall value. As a result, we selected specification 3 for the remainder of the analysis.

### ***Appendix 3.2.2. Main analysis and sensitivity tests***

We observed that the predictor means for Glasgow were closely aligned with those of the synthetic Glasgow, compared to the average of all donor pool/control cities (Table SC 4). Per capita gross disposable household income (63%) and the number of on-premises alcohol outlets (37%) emerged as the most influential covariates in generating the synthetic control weights. Among the donor pool cities, Dundee and North Ayrshire were the most significant contributors to the creation of synthetic Glasgow (Figure SC 06).

For Glasgow, we estimated a positive average treatment effect (ATT) of 0·047. However, the effect sizes estimated using ARIMA models were negative, indicating a discrepancy between the synthetic control and ARIMA approaches. Additionally, the p-value from the placebo test was 0·926, indicating a high level of uncertainty (Figure SC 10a). When we further modelled ARIMA with synthetic control, we estimated negative and statistically significant effect sizes (Table A17). Despite the different nature of p-values (inferential for ARIMA and frequentist for Synthetic) the different results from different approaches in terms of direction and significance weakened our confidence in interpreting the overall impact of the policy change in Glasgow, making these results inconclusive.

| **Table SC 3. Validation test for synthetic control for alcohol-related ambulance call-outs in Glasgow** | | | |
| --- | --- | --- | --- |
| **Specifications** | **Pre-intervention periods split between training and validation** | | |
|  | **Training/validation, 50/50** | **Training/validation, 70/30** | **Training/validation, 80/20** |
|  | **MSPE* (Validation)** | **MSPE* (Validation)** | **MSPE* (Validation)** |
| 1. (all pre-intervention outcome values and covariates) | 0·0618 | 0·05474 | 0·0662 |
| 2. (all pre-intervention outcome values only) | 0·0618 | 0·05474 | 0·0662 |
| 3. (pre-intervention outcome mean and covariates) | 0·0604 | 0·0667 | 0·0489 |
| 4. (last pre-intervention outcome value and covariates) | 0·0810 | 0·0706 | 0·0605 |
| 5. (pre-intervention outcome mean and last pre-intervention outcome value, and covariates) | 0·0604 | 0·0532 | 0·0729 |
| 6. (first three-fourths of the pre-intervention outcome values and covariates) | 0·0643 | 0·0606 | 0·0662 |
| 7. (last three-fourths of the pre-intervention outcome values and covariates) | 0·0613 | 0·0531 | 0·0620 |
| 8. (odd pre-intervention outcome values and covariates) | 0·0618 | 0·0547 | 0·0662 |
| 9. (even pre-intervention outcome values and covariates) | 0·0618 | 0·0547 | 0·0662 |

**Glasgow- Synthetic Glasgow, Specification 3:**

| **Table SC 4. Covariate balance in the pre-intervention periods** | | | | |  | |
| --- | --- | --- | --- | --- | --- | --- |
| **Covariates** | **Covariate weights** | **Alcohol-related ambulance call-outs, predictor means** | | | |  |
|  |  | **Glasgow** | **Synthetic Glasgow** | **Average of other control cities** | |  |
| Number of on-premises alcohol outlets (population size adjusted) | 0·370 | 21·416 | 21·446 | 23·524 | |  |
| Per capita gross disposable household income | 0·628 | 15852 | 15851 | 18260 | |  |
| Pre-intervention outcome mean | 0·002 | 0·964 | 0·835 | 0·595 | |  |

**Figure SC 06: Optimal unit weights**


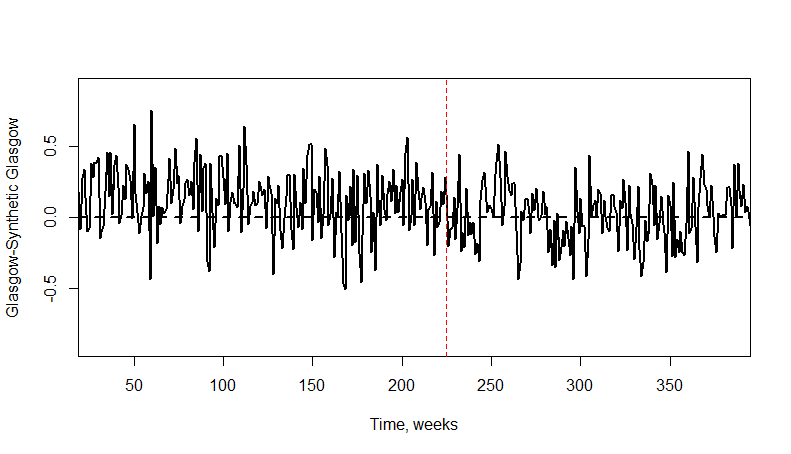


**Figure SC 07: Gaps of Glasgow and synthetic Glasgow**

**Figure SC 08a: Placebo test**

**Figure SC 08b: Placebo test excluding control cities MSPE>2 times Glasgow**

**Figure SC 08c: Placebo test excluding control cities if MSPE> Glasgow**

**Figure SC 09a: Effect size for placebo test**

**Figure SC 09b: Effect size placebo test considering only positive effect size**

**Figure SC 10a:** **Post/Pre MSPE ratio across cities**

**Figure SC 10b: Post/Pre MSPE ratio across cities considering only positive effect size**

## **Appendix 3.3. Reported crimes (Aberdeen)**

### ***Appendix 3.3.1. Model specification and validation test***

For the reported crimes in Aberdeen, we identified three specifications (6, 7, and 9) that generated the lowest MSPEs (Table SC 5). Specification 6 had the lowest MSPE value among them. As a result, we selected specification 6 for the remainder of the analysis.

### ***Appendix 3.3.2. Main analysis and sensitivity tests***

We observed that the predictor means for Aberdeen were closely aligned with those of the synthetic Aberdeen with some exceptions (Table SC 6). Among the donor pool cities, Dundee, Edinburgh, West Lothian, and Falkirk were the most significant contributors to the creation of synthetic Aberdeen for reported crimes (Figure SC 11).

We estimated a positive average treatment effect (ATT) of 0·013, consistent with the direction of the effect sizes estimated using ARIMA based on difference-in-Differences approaches. However, the p-value from the placebo test for the synthetic control was 0·444 (Figure SC 15a). When we further modelled ARIMA with synthetic control, we also estimated a negative but statistically insignificant effect size of -0.069 (Table A18). Although the effect sizes across different approaches (ARIMA and synthetic control) were homogeneous in terms of direction (positive), they were not always statistically significant. This underscores the uncertainty in the results and the need for caution in their interpretation.

| **Table SC 5. Validation test for synthetic control for reported crimes in Aberdeen** | | | |
| --- | --- | --- | --- |
| **Specifications** | **Pre-intervention periods split between training and validation** | | |
|  | **Training/validation, 50/50** | **Training/validation, 70/30** | **Training/validation, 80/20** |
|  | **MSPE* (Validation)** | **MSPE* (Validation)** | **MSPE* (Validation)** |
| 1. (all pre-intervention outcome values and covariates) | 0·0772 | 0·05798 | 0·0570 |
| 2. (all pre-intervention outcome values only) | 0·0767 | 0·05798 | 0·0570 |
| 3. (pre-intervention outcome mean and covariates) | 0·0724 | 0·0639 | 0·0665 |
| 4. (last pre-intervention outcome value and covariates) | 0·1128 | 0·2336 | 0·0729 |
| 5. (pre-intervention outcome mean and last pre-intervention outcome value, and covariates) | 0·0722 | 0·0640 | 0·0682 |
| 6. (first three-fourths of the pre-intervention outcome values and covariates) | 0·0858 | 0·0714 | 0·0534 |
| 7. (last three-fourths of the pre-intervention outcome values and covariates) | 0·0707 | 0·0640 | 0·0693 |
| 8. (odd pre-intervention outcome values and covariates) | 0·0745 | 0·0647 | 0·0565 |
| 9. (even pre-intervention outcome values and covariates) | 0·0772 | 0·0570 | 0·0556 |

**Aberdeen- Synthetic Aberdeen, Specification 6:**

| **Table SC 6. Covariate balance in the pre-intervention periods** | | | | |  | | |
| --- | --- | --- | --- | --- | --- | --- | --- |
| **Covariates** | **Covariate weights** | **Reported crimes, predictor means** | | | | |  |
|  |  | **Aberdeen** | **Synthetic Aberdeen** | **Average of other control cities** | |  |  |
| Number of on-premises alcohol outlets (population size adjusted) | 0·043 | 19·794 | 20·535 | 23·558 | |  |  |
| Per capita gross disposable household income | 0·001 | 20125 | 17415 | 17773 | |  |  |
| Pre-intervention outcome, period-19 | 0·026 | 0·695 | 0·702 | 0·655 | |  |  |
| Pre-intervention outcome, period-20 | 0·000 | 0·825 | 0·976 | 0·534 | |  |  |
| Pre-intervention outcome, period-21 | 0·004 | 0·825 | 0·773 | 0·749 | |  |  |
| Pre-intervention outcome, period-22 | 0·103 | 1·086 | 0·895 | 0·677 | |  |  |
| Pre-intervention outcome, period-23 | 0·004 | 0·608 | 0·720 | 0·641 | |  |  |
| Pre-intervention outcome, period-24 | 0·000 | 0·825 | 0·926 | 0·695 | |  |  |
| Pre-intervention outcome, period-25 | 0·040 | 0·565 | 0·679 | 0·764 | |  |  |
| Pre-intervention outcome, period-26 | 0·019 | 0·825 | 0·902 | 0·644 | |  |  |
| Pre-intervention outcome, period-27 | 0·030 | 0·738 | 0·700 | 0·674 | |  |  |
| Pre-intervention outcome, period-28 | 0·001 | 0·434 | 0·733 | 0·574 | |  |  |
| Pre-intervention outcome, period-29 | 0·081 | 0·695 | 0·667 | 0·598 | |  |  |
| Pre-intervention outcome, period-30 | 0·001 | 0·739 | 0·884 | 0·642 | |  |  |
| Pre-intervention outcome, period-31 | 0·020 | 1·260 | 1·086 | 0·905 | |  |  |
| Pre-intervention outcome, period-32 | 0·017 | 0·565 | 0·966 | 0·765 | |  |  |
| Pre-intervention outcome, period-33 | 0·013 | 0·695 | 0·872 | 0·777 | |  |  |
| Pre-intervention outcome, period-34 | 0·047 | 0·826 | 0·783 | 0·595 | |  |  |
| Pre-intervention outcome, period-35 | 0·044 | 1·478 | 0·852 | 0·716 | |  |  |
| Pre-intervention outcome, period-36 | 0·062 | 0·826 | 0·691 | 0·642 | |  |  |
| Pre-intervention outcome, period-37 | 0·005 | 0·435 | 0·712 | 0·668 | |  |  |
| Pre-intervention outcome, period-38 | 0·047 | 0·869 | 1·028 | 0·631 | |  |  |
| Pre-intervention outcome, period-39 | 0·116 | 1·043 | 0·912 | 0·663 | |  |  |
| Pre-intervention outcome, period-40 | 0·078 | 0·696 | 0·677 | 0·545 | |  |  |
| Pre-intervention outcome, period-41 | 0·094 | 1·261 | 0·962 | 0·544 | |  |  |
| Pre-intervention outcome, period-42 | 0·097 | 0·870 | 0·725 | 0·724 | |  |  |
| Pre-intervention outcome, period-43 | 0·007 | 1·044 | 0·632 | 0·592 | |  |  |

**Figure SC 11: Optimal unit weights**

**Figure SC 12: Gaps of Aberdeen and synthetic Aberdeen**

**Figure SC 13a: Placebo test**

**Figure SC 13b: Placebo test excluding control cities MSPE>2 times Aberdeen**

**Figure SC 13c: Placebo test excluding control cities if MSPE> Aberdeen**

**Figure SC 14a: Effect size for placebo test**

**Figure SC 14b: Effect size placebo test considering only positive effect size**

**Figure SC 15a: Post/Pre MSPE ratio across cities**

**Figure SC 15b: Post/Pre MSPE ratio across cities considering only positive effect size**

## **Appendix 3.4. Reported crimes (Glasgow)**

### ***Appendix 3.4.1. Model specification and validation test***

For the reported crimes in Glasgow, we identified specifications 6, 7, and 9 that generated the lowest MSPEs (Table SC 7). Among them specification 7 a had the lowest value of MSPE. As a result, we selected specification 7 for the remainder of the analysis.

### ***Appendix 3.4.2. Main analysis and sensitivity tests***

We observed that the predictor means for Glasgow were closely aligned with those of the synthetic Glasgow with some exceptions (Table SC 8). Among the donor pool cities, Edinburgh, Dundee, and Falkirk were the most significant contributors to the creation of synthetic Glasgow for reported crimes (Figure SC 11).

We estimated a positive average treatment effect (ATT) of 0·133. However, the effect sizes estimated using ARIMA based on difference-in-Differences models were negative and insignificant, indicating a discrepancy between the synthetic control and ARIMA approaches. Additionally, the p-value from the placebo test was 0·926, indicating a high level of uncertainty (Figure SC 20a). When we further modelled ARIMA with synthetic control, we estimated negative and statistically insignificant effect size (Table A18). Despite the different nature of p-values (inferential for ARIMA and frequentist for Synthetic) the different results from different approaches in terms of direction and significance weakened our confidence in interpreting the overall impact of the policy change in Glasgow, making these results unconclusive.

| **Table SC 7. Validation test for synthetic control for reported crimes in Glasgow** | | | |
| --- | --- | --- | --- |
| **Specifications** | **Pre-intervention periods split between training and validation** | | |
|  | **Training/validation, 50/50** | **Training/validation, 70/30** | **Training/validation, 80/20** |
|  | **MSPE* (Validation)** | **MSPE* (Validation)** | **MSPE* (Validation)** |
| 1. (all pre-intervention outcome values and covariates) | 0·0765 | 0·05166 | 0·0473 |
| 2. (all pre-intervention outcome values only) | 0·0765 | 0·05133 | 0·0473 |
| 3. (pre-intervention outcome mean and covariates) | 0·0853 | 0·0819 | 0·0632 |
| 4. (last pre-intervention outcome value and covariates) | 0·1298 | 0·0670 | 0·0598 |
| 5. (pre-intervention outcome mean and last pre-intervention outcome value, and covariates) | 0·0930 | 0·0643 | 0·0584 |
| 6. (first three-fourths of the pre-intervention outcome values and covariates) | 0·0745 | 0·0513 | 0·0473 |
| 7. (last three-fourths of the pre-intervention outcome values and covariates) | 0·0724 | 0·0513 | 0·0473 |
| 8. (odd pre-intervention outcome values and covariates) | 0·0766 | 0·0517 | 0·0473 |
| 9. (even pre-intervention outcome values and covariates) | 0·0724 | 0·0513 | 0·0475 |

**Glasgow- Synthetic Glasgow, Specification 7:**

| **Table SC 8. Covariates balance in the pre-intervention periods** | | | |  |
| --- | --- | --- | --- | --- |
| **Covariates** | **Covariate weights** | **Reported crimes, predictor means** | | |
|  |  | **Glasgow** | **Synthetic Glasgow** | **Average of other control cities** |
| Number of on-premises alcohol outlets (population size adjusted) | 0·016 | 21·371 | 23·402 | 23·529 |
| Per capita gross disposable household income | 0·008 | 15852 | 18900 | 18246 |
| Pre-intervention outcome, period-169 | 0·019 | 0·733 | 0·835 | 0·748 |
| Pre-intervention outcome, period-170 | 0·017 | 0·701 | 0·720 | 0·456 |
| Pre-intervention outcome, period-171 | 0·012 | 0·637 | 0·701 | 0·413 |
| Pre-intervention outcome, period-172 | 0·011 | 0·987 | 1·085 | 0·539 |
| Pre-intervention outcome, period-173 | 0·017 | 1·002 | 0·779 | 0·543 |
| Pre-intervention outcome, period-174 | 0·015 | 1·114 | 0·676 | 0·519 |
| Pre-intervention outcome, period-175 | 0·005 | 0·891 | 0·411 | 0·421 |
| Pre-intervention outcome, period-176 | 0·026 | 0·954 | 0·811 | 0·516 |
| Pre-intervention outcome, period-177 | 0·016 | 0·922 | 0·964 | 0·567 |
| Pre-intervention outcome, period-178 | 0·014 | 0·763 | 0·398 | 0·501 |
| Pre-intervention outcome, period-179 | 0·021 | 0·938 | 0·681 | 0·495 |
| Pre-intervention outcome, period-180 | 0·020 | 0·826 | 0·589 | 0·470 |
| Pre-intervention outcome, period-181 | 0·014 | 0·826 | 0·618 | 0·468 |
| Pre-intervention outcome, period-182 | 0·014 | 1·080 | 1·178 | 0·795 |
| Pre-intervention outcome, period-183 | 0·006 | 0·889 | 0·545 | 0·525 |
| Pre-intervention outcome, period-184 | 0·017 | 0·826 | 0·798 | 0·556 |
| Pre-intervention outcome, period-185 | 0·020 | 0·730 | 0·636 | 0·458 |
| Pre-intervention outcome, period-186 | 0·015 | 0·746 | 0·855 | 0·562 |
| Pre-intervention outcome, period-187 | 0·016 | 0·793 | 0·717 | 0·505 |
| Pre-intervention outcome, period-188 | 0·014 | 0·809 | 0·622 | 0·523 |
| Pre-intervention outcome, period-189 | 0·017 | 0·682 | 0·920 | 0·565 |
| Pre-intervention outcome, period-190 | 0·006 | 0·682 | 0·857 | 0·528 |
| Pre-intervention outcome, period-191 | 0·024 | 1·094 | 0·723 | 0·597 |
| Pre-intervention outcome, period-192 | 0·018 | 0·824 | 0·690 | 0·585 |
| Pre-intervention outcome, period-193 | 0·020 | 0·713 | 0·535 | 0·401 |
| Pre-intervention outcome, period-194 | 0·018 | 0·681 | 0·573 | 0·419 |
| Pre-intervention outcome, period-195 | 0·016 | 0·776 | 0·804 | 0·539 |
| Pre-intervention outcome, period-196 | 0·023 | 0·792 | 0·969 | 0·543 |
| Pre-intervention outcome, period-197 | 0·017 | 0·586 | 0·516 | 0·566 |
| Pre-intervention outcome, period-198 | 0·027 | 0·886 | 0·575 | 0·629 |
| Pre-intervention outcome, period-199 | 0·020 | 0·918 | 0·950 | 0·591 |
| Pre-intervention outcome, period-200 | 0·018 | 1·013 | 0·962 | 0·528 |
| Pre-intervention outcome, period-201 | 0·017 | 0·823 | 0·822 | 0·475 |
| Pre-intervention outcome, period-202 | 0·013 | 0·854 | 0·694 | 0·552 |
| Pre-intervention outcome, period-203 | 0·018 | 0·522 | 0·468 | 0·370 |
| Pre-intervention outcome, period-204 | 0·028 | 1·360 | 0·913 | 0·616 |
| Pre-intervention outcome, period-205 | 0·022 | 0·711 | 0·666 | 0·459 |
| Pre-intervention outcome, period-206 | 0·013 | 0·616 | 0·794 | 0·563 |
| Pre-intervention outcome, period-207 | 0·020 | 1·043 | 0·720 | 0·595 |
| Pre-intervention outcome, period-208 | 0·018 | 0·964 | 0·702 | 0·594 |
| Pre-intervention outcome, period-209 | 0·020 | 0·521 | 0·469 | 0·267 |
| Pre-intervention outcome, period-210 | 0·021 | 0·805 | 0·447 | 0·344 |
| Pre-intervention outcome, period-211 | 0·036 | 0·758 | 0·527 | 0·341 |
| Pre-intervention outcome, period-212 | 0·013 | 1·074 | 0·562 | 0·413 |
| Pre-intervention outcome, period-213 | 0·016 | 0·932 | 0·950 | 0·529 |
| Pre-intervention outcome, period-214 | 0·016 | 0·774 | 0·622 | 0·557 |
| Pre-intervention outcome, period-215 | 0·011 | 0·821 | 0·563 | 0·410 |
| Pre-intervention outcome, period-216 | 0·022 | 0·868 | 0·726 | 0·476 |
| Pre-intervention outcome, period-217 | 0·012 | 1·026 | 0·608 | 0·496 |
| Pre-intervention outcome, period-218 | 0·020 | 0·884 | 0·617 | 0·559 |
| Pre-intervention outcome, period-219 | 0·021 | 0·758 | 0·512 | 0·483 |
| Pre-intervention outcome, period-220 | 0·018 | 0·852 | 0·630 | 0·480 |
| Pre-intervention outcome, period-221 | 0·018 | 0·947 | 0·900 | 0·507 |
| Pre-intervention outcome, period-222 | 0·019 | 0·458 | 0·597 | 0·521 |
| Pre-intervention outcome, period-223 | 0·014 | 0·647 | 0·422 | 0·401 |
| Pre-intervention outcome, period-224 | 0·017 | 0·883 | 0·706 | 0·422 |

**Figure SC 16: Optimal unit weights**

**Figure SC 17: Gaps of Glasgow and synthetic Glasgow**

**Figure SC 18a: Placebo test**

**Figure SC 18b: Placebo test excluding control cities MSPE>2 times Glasgow**

**Figure SC 18c: Placebo test excluding control cities if MSPE> Glasgow**

**Figure SC 19a: Effect size for placebo test**

**Figure SC 19b: Effect size placebo test considering only positive effect size**

**Figure SC 20a: Post/Pre MSPE ratio across cities**

**Figure SC 20b: Post/Pre MSPE ratio across cities considering only positive effect size**

# **Appendix 4. Synthetic control and ARIMA**

| **Table A17. Effect of policy changes on weekend night-time alcohol-related ambulance call-outs in Aberdeen and Glasgow** | | | |
| --- | --- | --- | --- |
| **Primary outcome, weekend night-time alcohol-related ambulance callouts** | **Synthetic control + ARIMA** | | |
|  | **Outcome: population adjusted incident rates** | | |
|  | **Coefficient** | **P-value** | **95% CI** |
| **Intervention area: Aberdeen** | | | |
| **Staggered policy covariate** |  |  |  |
| Policy effect (all covariates)* | **0.205** | 0.000 | 0.106, 0.305 |
| Policy effect (significant covariates) | **0.205** | 0.000 | 0.105, 0.305 |
| **Dummy policy covariate, sensitivity analysis** |  |  |  |
| Policy effect (all covariates) | **0.146** | 0.000 | 0.078, 0.213 |
| Policy effect (significant covariates) | **0.146** | 0.000 | 0.079, 0.214 |
| **Dummy policy covariate with policy implemented with at least half strength, sensitivity analysis** |  |  |  |
| Policy effect (all covariates) | **0.101** | 0.005 | 0.030, 0.172 |
| Policy effect (significant covariates) | **0.100** | 0.006 | 0.029, 0.171 |
| **Intervention area: Glasgow** | | | |
| **Dummy policy covariate** |  |  |  |
| Policy effect (all covariates)* | **-0.080** | 0.011 | -0.141, -0.018 |
| Policy effect (significant covariates) | **-0.080** | 0.011 | -0.141, -0.019 |
| *Models are adjusted by covariates- public holidays and outliers | | | |

| **Table A18. Effect of policy changes on weekend night-time recorded crimes in Aberdeen and Glasgow** | | | |
| --- | --- | --- | --- |
| **Secondary outcome, weekend night-time recorded crimes** | **Synthetic control + ARIMA** | | |
|  | **Outcome: population adjusted incident rates** | | |
|  | **Coefficient** | **P-value** | **95% CI** |
| **Intervention area: Aberdeen** | | | |
| **Staggered policy covariate** |  |  |  |
| Policy effect (all covariates)* | -0.067 | 0.213 | -0.173, 0.039 |
| Policy effect (significant covariates) | -0.069 | 0.201 | -0.175, 0.037 |
| **Dummy policy covariate, sensitivity analysis** |  |  |  |
| Policy effect (all covariates) | -0.052 | 0.123 | -0.119, 0.014 |
| Policy effect (significant covariates) | -0.052 | 0.125 | -0.119, 0.015 |
| **Dummy policy covariate with policy implemented with at least half strength, sensitivity analysis** |  |  |  |
| Policy effect (all covariates) | 0.003 | 0.943 | -0.084, 0.090 |
| Policy effect (significant covariates) | 0.001 | 0.983 | -0.085, 0.087 |
| **Intervention area: Glasgow** | | | |
| **Dummy policy covariate** |  |  |  |
| Policy effect (all covariates)* | -0.030 | 0.326 | -0.089, 0.030 |
| Policy effect (significant covariates) | -0.030 | 0.324 | -0.089, 0.029 |
| *Models are adjusted by covariates- public holidays and outliers | | | |

**References**

1 Scottish Government. Quarterly National Accounts Scotland, 2017 Quarter 3. 2018. https://www.webarchive.org.uk/wayback/archive/20180212144444/http://www.gov.scot/Topics/Statistics/Browse/Economy/QNA2017Q3 (accessed Nov 22, 2022).

2 Centre for Environmental Data Analysis. Met Office weather datasets. CEDA Arch. 2019. https://data.ceda.ac.uk/badc/ukmo-hadobs/data/insitu/MOHC/HadOBS/HadUK-Grid/v1.2.0.ceda/region (accessed Nov 10, 2023).

3 The Scottish Government. Scottish Liquor Licensing Statistics. 2022. https://www.gov.scot/publications/scottish-liquor-licensing-statistics/.

4 Scottish Parliament Information Centre (SPICe). Timeline of Coronavirus (COVID-19) in Scotland. SPICe Spotlight. 2023. https://spice-spotlight.scot/2023/05/10/timeline-of-coronavirus-covid-19-in-scotland/ (accessed March 11, 2024).

5 The Scottish Government. Public and bank holidays. Scotl. bank holidays. 2022. https://www.mygov.scot/scotland-bank-holidays (accessed March 12, 2024).

6 Abadie A, Vives-i-Bastida J. Synthetic Controls in Action. *arXiv Prepr* 2022. http://arxiv.org/abs/2203.06279.

7 Ferman B, Pinto C, Possebom V. Cherry Picking with Synthetic Controls. *J Policy Anal Manag* 2020; **39**: 510–32.

8 Kaul A, Klößner S, Pfeifer G, Schieler M. Standard Synthetic Control Methods: The Case of Using All Preintervention Outcomes Together With Covariates. *J Bus Econ Stat* 2022; **40**: 1282–90.

9 Kreif N, Grieve R, Hangartner D, Turner AJ, Nikolova S, Sutton M. Examination of the synthetic control method for evaluating health policies with multiple treated units. *Health Econ* 2015; **25**: 1514–28.
